# Coupling Vgll4b/Yap-regulated posterior cell addition with anterior vacuolation enables robust notochord elongation

**DOI:** 10.1242/dev.205951

**Published:** 2026-09-14

**Authors:** Carlos Camacho-Macorra, Alberto S. Ceccarelli, Guillermo Serrano Nájera, Dillan Saunders, Isabella Boesgaard, Osvaldo Chara, Benjamin Steventon

**Affiliations:** ^1^ Department of Genetics, University of Cambridge, Cambridge, CB2 3EH, UK; ^2^ Selwyn College, University of Cambridge, Cambridge, CB3 9DQ, UK; ^3^ School of Biosciences, University of Nottingham, Nottingham, LE12 5RD, UK; ^4^ Instituto de Tecnología, Universidad Argentina de la Empresa, Buenos Aires C1073AAO, Argentina

**Keywords:** Axis extension, Tailbud, Progenitor addition, Zebrafish, Modelling, Morphogenesis

## Abstract

Robust tissue growth control requires long-range communication between the rate of progenitor addition and tissue expansion. However, the regulatory mechanisms that couple these processes are unknown. In zebrafish, notochord morphogenesis is a driver of axis extension through both posterior progenitor addition and anterior vacuolation. To elucidate how progenitor dynamics and vacuole-driven cell expansion interact to elongate the notochord, we generated a mathematical model linking progenitor addition rate to the expansion of cells from anterior to posterior to simulate vacuolation rate. Comparing this with empirical measurements, we find that progenitor incorporation, together with vacuolation, produces a linear gradient in nearest neighbour distance. We next explored the role of YAP/TAZ in regulating progenitor addition in mutants for the YAP/TAZ inhibitor *vgll4b*. We find that *vgll4b* expression and YAP activity are enriched in posterior midline progenitors. Loss of *vgll4b* elevates YAP signalling, enhances progenitor addition, restricts vacuole expansion, and – after a transient buffering phase – compromises anterior-posterior axis elongation. These results support a long-range feedback mechanism linking progenitor recruitment to vacuolation, enabling the notochord to balance cellular input with volumetric expansion, thereby maintaining tissue proportions.

## Teaser

**Highlighted Article:** YAP activation in notochord progenitors of *vgll4b* mutant zebrafish leads to enhanced progenitor addition to the notochord, compromising the ability of notochord cells to undergo vacuolation, resulting in reduced axial elongation.

## INTRODUCTION

A defining feature of vertebrate development is the elongation of the body along the anterior–posterior (A–P) axis, a process in which the generation of new tissue at the posterior end is coordinated with differentiation and growth in more anterior regions (Bénazéraf et al., 2017; Mongera et al., 2019; Steventon et al., 2016). This process requires the integrated action of cellular, molecular and mechanical mechanisms across spatially distinct regions of the embryo to maintain proportionate axis elongation. Multiple, partially overlapping mechanisms contribute to vertebrate axis extension. During gastrulation and early somitogenesis, convergent extension movements narrow and lengthen the embryonic body plan (Keller, 2002; Steventon et al., 2016). Subsequently, distinct populations of progenitors in the tailbud supply cells to the forming neural tube, notochord and somites (Wymeersch et al., 2021). In parallel, mechanical forces arising from tissue tension, extracellular matrix remodelling and hydrostatic pressure shape and elongate axial structures (Adams et al., 1990; Irvine and Shraiman, 2017; Michaut et al., 2025; Mongera et al., 2018). Furthermore, mechanical and spatial interactions between adjacent tissues regulate tissue proportions through a phenomenon referred to as multi-tissue tectonics (Blanchard et al., 2009; Busby and Steventon, 2021; Saunders et al., 2025). Because these processes occur concurrently and mutually influence one another, embryos must coordinate them to ensure robust patterning and preserve axial proportions (Saiz and Hadjantonakis, 2020). Despite extensive work on the cellular sources and molecular signals that govern posterior axis formation, the mechanisms by which embryos balance posterior tissue generation with anterior tissue expansion remain incompletely understood.

This mechanism is particularly relevant for structures such as the zebrafish notochord, a defining feature of the chordate body plan. In addition to providing physical support and patterning cues to surrounding tissues (Halpern, 1997; Stemple, 2005), the notochord plays a central role in mechanically elongating the A–P axis (Adams et al., 1990; McLaren and Steventon, 2021). In zebrafish, midline progenitor cells located in the basal region of the tailbud surrounding the chordoneural hinge give rise to the mesodermal notochord, neural floor plate and endodermal hypochord lineages (Row et al., 2016). The hypochord is a transient endodermal structure present in teleosts and some other vertebrates, positioned ventral to the notochord and contributing to the patterning of surrounding tissues (Hogan and Bautch, 2004).

**Fig. 1.**
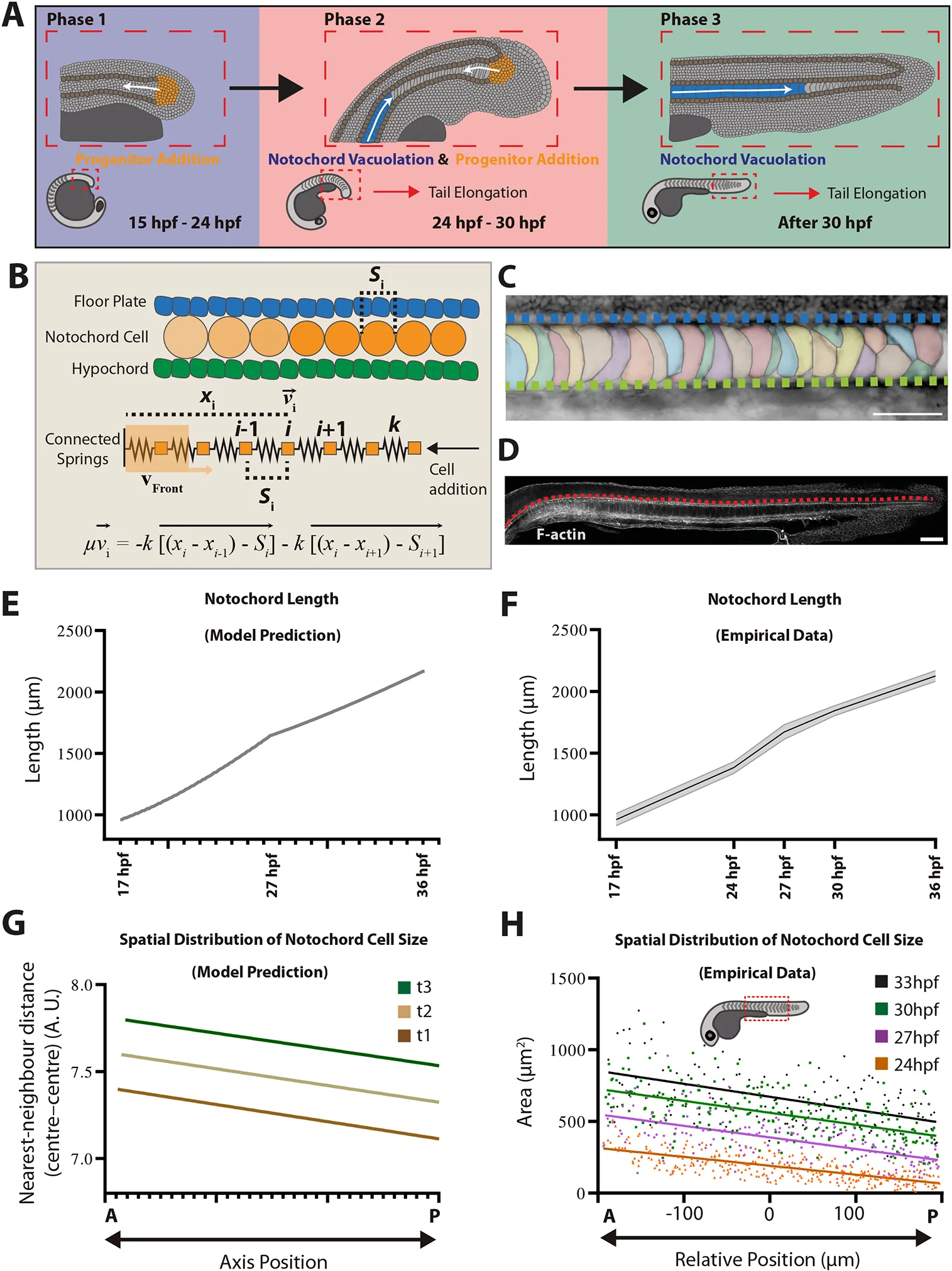
**Mathematical model predicting notochord elongation dynamics and scaling properties in zebrafish embryos.** (A) Scheme representing the three phases of late notochord morphogenesis. The last two phases are described in the mathematical model. White arrows denote cell movements. Colors indicate the notochord progenitor niche (orange), vacuolated notochord cells (blue), tissues flanking the notochord, floor plate and hypochord (brown), and surrounding embryonic tissues (grey). (B) Scheme illustrating key concepts used to construct the mathematical model. A schematic of the tissue (top), the associated physical model of coupled spring and masses (middle) and the force balance at each mass (bottom). (C) Examples of notochord vacuole segmentation visualized with BODIPY staining; the blue line marks the notochord and the green line marks the hypochord. Scale bar: 50 μm. (D) Example of the methodology used to measure notochord length by tracing the floor plate (red line) in confocal images of phalloidin-stained embryos. Scale bar: 100 μm. (E) Mathematical model prediction of notochord length at different time points. (F) Quantification of A–P axis length at 17 hpf (*n*=12), 24 hpf (*n*=10), 27 hpf (*n*=20), 30 hpf (*n*=16) and 36 hpf (*n*=7). (G) Mathematical model prediction of AP profiles of centre-to-centre nearest-neighbour cell distances within the notochord at different time points. A.U., arbitrary units. (H) Quantification of cell area within the notochord at 24 hpf, 27 hpf, 30 hpf and 33 hpf. The A–P axis in the graph is centred at the yolk-level extension. The ROI used for measurements spanned 200 μm on each side of this reference point, for a total length of 400 μm. Solid lines represent the best linear fits.

Zebrafish notochord morphogenesis can be thought of in terms of three distinct phases (Fig. 1A). In the first phase, posterior progenitor addition drives elongation by incorporating new cells into the posterior notochord. In the second phase, progenitor addition occurs together with the anterior vacuolation causes differentiated notochord cells to increase in volume, creating a ‘vacuolation front’ that progresses from anterior to posterior along the axis. The resulting pressure generates mechanical forces that stretch and elongate the notochord (Ellis et al., 2013; McLaren and Steventon, 2021; Stemple, 2005). Upon progenitor depletion, a final phase is characterized by continued notochord vacuolation at post-somitogenesis stages of development. Achieving the correct balance between these opposing dynamics is essential for producing a properly proportioned notochord and, by extension, a correctly scaled vertebrate body axis (McLaren and Steventon, 2021). Disruption of either progenitor addition (Talbot et al., 1995) or notochord cell vacuolation (Bagwell et al., 2020; Ellis et al., 2013) results in a shortened A–P axis. Progenitor addition is a key determinant of notochord elongation and cell number, yet insights into the rate of incorporation and the molecular pathways regulating the process remain limited. Understanding how progenitor recruitment is coordinated with tissue-scale processes, such as anterior vacuolation, is essential to elucidate the mechanisms that ensure robust notochord elongation and proper A–P scaling in the zebrafish embryo.

The Hippo pathway effector YAP, through its interaction with TEAD transcription factors, has emerged as a central regulator of mechanotransduction (Dupont et al., 2011), progenitor maintenance (Han et al., 2015; Wang et al., 2016) and axial development (Porazinski et al., 2015) across vertebrate species. Targeted disruption of Yap in mouse models leads to severe perturbations in embryonic body axis elongation (Morin-Kensicki et al., 2006). YAP expression has been documented in the notochord across multiple vertebrate models, including mouse (Sawada et al., 2008), frog (Nejigane et al., 2011) and zebrafish (Jiang et al., 2009), indicating an evolutionarily conserved presence in this axial structure. Recent cross-species and stem cell studies further suggest that YAP plays a functional role in axis specification and notochord development. In human embryonic stem cell-derived gastruloids and organoids, YAP1 regulates self-organized germ layer fate patterning and axis morphogenetic events (Rito et al., 2025; Stronati et al., 2022), while in mouse embryos YAP responds to mechanical inputs to regulate FoxA2 and Shh expression, thereby coordinating notochord and floor plate formation (Cheng et al., 2023). In medaka, YAP drives a mechanosensitive programme that sustains the collective cell migration required for axis assembly (Sousa-Ortega et al., 2023). In zebrafish, *yap1* and its paralogue *wwtr1* are expressed and active in the notochord (Astone et al., 2024; Kimelman et al., 2017) and contribute to posterior body elongation and overall morphogenesis (Kimelman et al., 2017). Despite these insights, the specific roles of YAP in the morphogenesis of the zebrafish notochord and the mechanisms regulating its activity in this context remain incompletely defined.

VGLL4 is a well-established inhibitor of YAP activity (Tang et al., 2024; Zhu et al., 2025), acting through competitive binding to TEAD transcription factors (Deng and Fang, 2018; Shi et al., 2017; Zhang et al., 2021). This antagonism has led to the view that a major function of YAP in development is to counteract a default VGLL4-mediated repression (Cai et al., 2022). During embryogenesis, the VGLL4–TEAD axis regulates diverse processes, including cardiac development (Lin et al., 2016; Sheldon et al., 2022; Yu et al., 2019), skeletal muscle formation (Feng et al., 2019), left–right asymmetry (Fillatre et al., 2019), osteoblast differentiation (Suo et al., 2020) and posterior lateral line primordium migration (Lardennois et al., 2026). However, a potential role for VGLL4 in notochord morphogenesis has not yet been investigated.

To explore this possibility, we first developed a mathematical model integrating posterior progenitor addition and anterior vacuolation to investigate their combined effects on notochord elongation and cell size distribution. This computational framework allows us to explore how variations in progenitor incorporation rates or vacuolation dynamics influence tissue growth and morphogenesis. Importantly, the model provides a mechanistic basis to test our central hypothesis: that the balance between progenitor addition and vacuolation is actively regulated. By combining *in vivo* measurements, functional manipulations of YAP activity, and computational predictions, we aim to uncover the regulatory principles linking YAP signalling, progenitor behaviour and tissue-scale dynamics during vertebrate axis elongation.

## RESULTS

### Mathematical modelling reveals how posterior progenitor addition and anterior vacuolation drive notochord elongation and scaling of cell sizes

To investigate how posterior progenitor addition and cell vacuolation are coordinated during notochord elongation, we developed a minimal mathematical model representing the notochord as a linear sequence of mass–spring cells along the A–P axis, *x*. Similar biophysical and computational approaches have been used previously to study notochord elongation, cell packing, and morphogenetic mechanics in vertebrate embryos (Adams et al., 1990; Curcio and Lubkin, 2023; Yasuoka, 2020). In our model, progenitor cells are added with a rate *r_p_* at the posterior end at defined intervals by attaching to the terminal cell, while a vacuolation front advances posteriorly at constant speed *v*_*front*_, triggering linear growth (at a rate *J*) of target cell size *S*_*i*_ once cells lie anterior to the front (Fig. 1B-D). To improve our understanding of the coordination of these processes, we focused on the last two phases of notochord elongation: when progenitor addition coincides with vacuolation, and upon progenitor depletion when only vacuolation proceeds (Fig. 1A). A complete description of the model is provided in the Materials and Methods section.

As expected, the model predicts that both cell addition and vacuolation drive notochord elongation. Notably, the modelled notochord elongates approximately linearly in time during both phases, with comparable growth rates (Fig. 1E). To test this prediction, we measured notochord length in embryos at multiple time points between 17 and 36 h post fertilization (hpf) and observed a similar linear growth pattern (Fig. 1F). As vacuolation began anteriorly, cell sizes decreased linearly from anterior to posterior. Over time, this linear profile maintained its negative slope constant while its positive intercept increased (Fig. 1G). We refer to this phenotype as scaling, as the overall shape of the distribution is preserved during tissue growth. To test this prediction, we imaged and segmented notochords (Fig. 1C) spanning a 200 μm domain anterior and posterior to the yolk extension from 27 to 33 hpf and quantified the centre-to-centre nearest neighbour cell distances of notochord cells (Fig. S1A) as well as notochord cell area (Fig. 1H). Strikingly, the spatial profile of both cell distances and cell areas along the A–P axis were also linear, with a positive intercept that increased over time and a negative slope that remained nearly constant (Fig. 1H, Fig. S1A). Given that, in the zebrafish embryo, a single, large, fluid-filled vacuole occupies approximately 70-90% of the volume of notochord cells (Bagwell et al., 2020; Ellis et al., 2013), these findings raise the question of how the rate of progenitor addition is coordinated with vacuolation during notochord elongation.

### Active YAP and its inhibitor *vgll4b* colocalize with midline progenitors in the zebrafish tailbud

**Fig. 2.**
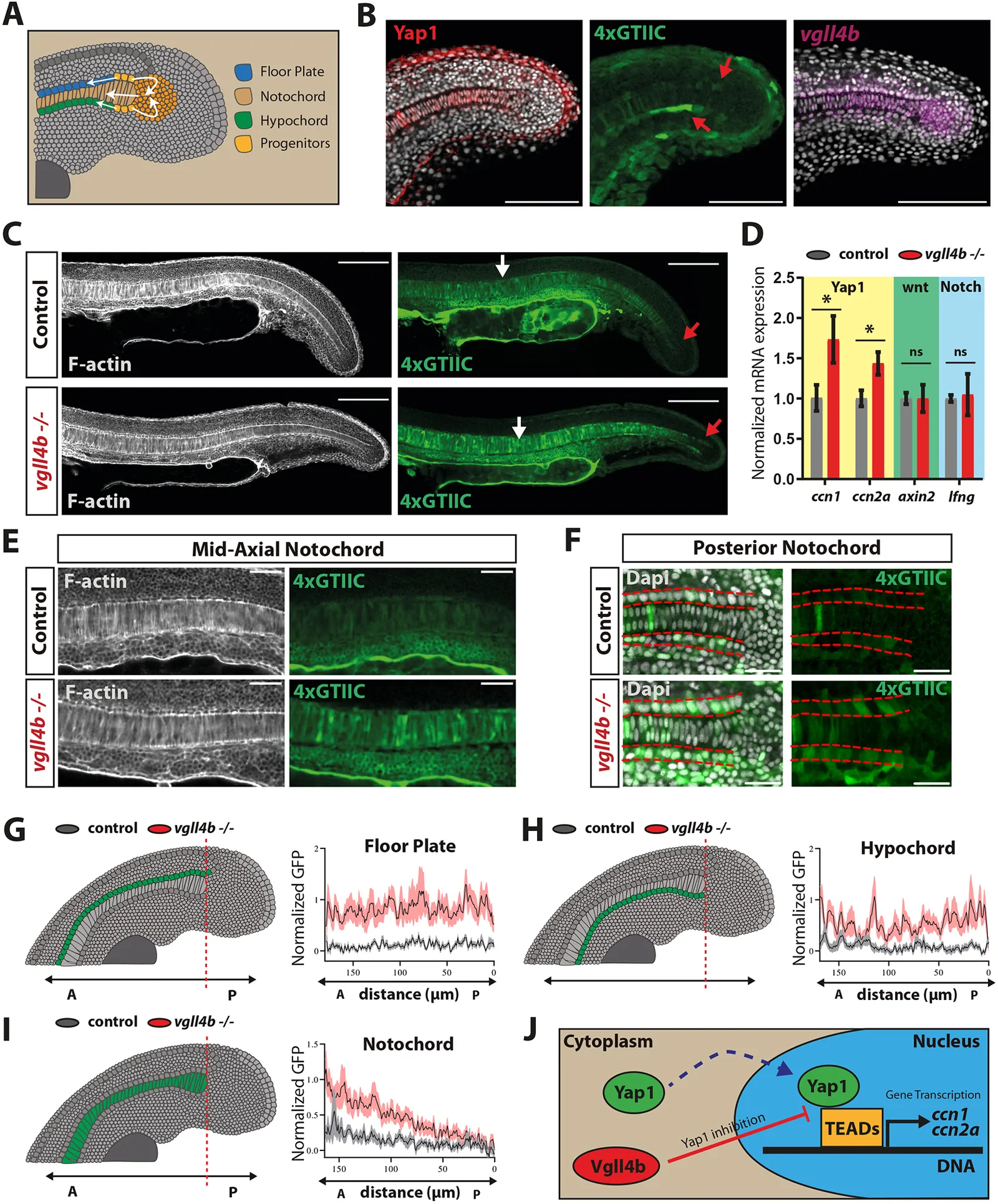
**YAP activity is observed in midline progenitors during axis elongation, and is upregulated in *vgll4b* mutants.** (A) Scheme representing different midline progenitor populations in the zebrafish tailbud. White arrows indicate the direction of cell movements. (B) Confocal images of 24 hpf *Tg(4xGTIIC:GFP)* embryos with immunostaining for YAP (Yap1; red) and GFP (green), and HCR for *vgll4b* mRNA (magenta). Scale bars: 100 μm. Red arrows point to GFP expression in the posterior floor plate and hypochord. (C) Confocal images of 24 hpf *Tg(4xGTIIC:GFP)* control and *Tg(4xGTIIC:GFP)*; *vgll4b*^−/−^ embryos. Red arrows point to GFP expression in the progenitor region and white arrows point to GFP expression in the anterior notochord. Scale bars: 100 μm. (D) qPCR analysis of 24 hpf tailbuds showing upregulation of the YAP targets *ccn1* (*t*-test, *P*=0.0305) and *ccn2a* (*t*-test, *P*=0.0157), while *axin2* (*t*-test, *P*=0.9985) and *lfng* (*t*-test, *P*=0.7785) remained unchanged (ns, not significant). (E) Confocal images of the mid-axial notochord in control and mutant embryos at 24 hpf. Scale bars: 50 μm. (F) Confocal images of the posterior notochord in control and mutant embryos at 24 hpf immunostained for GFP. Scale bars: 20 μm. The red dashed lines highlight the floor plate and the notochord. (G-I) Quantification of normalized GFP intensity across the floor plate, notochord and hypochord at 24 hpf, showing increased reporter activity in mutants (control *n*=13; mutant *n*=15). Red dashed lines indicate the posterior end of the notochord. In graphs, solid lines represent mean values and shaded areas denote data dispersion. (J) Scheme illustrating the Vgll4b–YAP inhibition mechanism. Cytoplasmic Yap1 translocates into the nucleus (dashed blue arrow), where it interacts with TEAD transcription factors to drive the expression of target genes (*ccn1* and *ccn2a*). Vgll4b inhibits Yap1-mediated transcriptional activity (red line).

The zebrafish tailbud contains distinct midline progenitor populations that give rise to mesodermal notochord, neural floor plate and endodermal hypochord lineages (Latimer and Appel, 2006; Row et al., 2016). Recent work has identified two previously uncharacterized midline progenitor pools located at the most posterior ends of the axial midline (Morabito et al., 2025 preprint; Row et al., 2016): a dorsal population at the posterior floor plate that generates both floor plate and notochord, and a ventral population at the posterior hypochord that contributes to hypochord and notochord (Fig. 2A). During notochord morphogenesis, these progenitors migrate posteriorly, and cells in the posterior-most region converge into a defined axial progenitor niche to form the extending notochord. In contrast, more anterior trailing progenitors gradually decelerate and differentiate into floor plate or hypochord, respectively.

To investigate factors that may regulate the rate at which notochord progenitors are incorporated into the posterior notochord, we focused on YAP, a mechanotransduction effector increasingly recognized as a key regulator of axial development (Sousa-Ortega et al., 2023). Using the YAP activity reporter line *4xGTIIC:GFP*, which marks cells with active YAP signalling (Miesfeld and Link, 2014), together with Yap1 immunostaining, we assessed Yap1 protein localization and activity in the tail during notochord morphogenesis. Consistent with previous findings (Kimelman et al., 2017), Yap1 protein was detected in the epidermis and in midline structures, including the notochord, floor plate and hypochord (Fig. 2B). Reporter activity highlighted strong YAP activation at the posterior ends of the floor plate and, most prominently, the hypochord (Fig. 2B), whereas more anterior regions showed clear activity also in the notochord (Fig. 2C,E). We next examined the gene expression of zebrafish Vgll4 paralogues during posterior axis elongation and found that *vgll4b* is the only paralogue expressed in the posterior tailbud (Fig. S1B). Its expression is localized to midline tissues, including the notochord, floor plate and hypochord, as well as to the region containing the earliest described notochord progenitors (Fig. 2B).

In summary, these observations demonstrate that YAP is actively engaged within the midline progenitor domain during zebrafish axis elongation, and that its inhibitor *vgll4b* is specifically expressed in the same axial tissues. This spatial overlap indicates that *vgll4b* could modulate YAP activity *in situ*, providing a mechanistic framework for how YAP signalling may regulate progenitor allocation and behaviour during notochord formation.

### *vgll4b* is required to limit YAP activity in the midline progenitors

To determine whether *vgll4b* regulates YAP activity during A–P axis elongation, we used a *vgll4b* loss-of-function line generated in the *4xGTIIC:GFP* reporter background (Camacho-Macorra et al., 2024). Comparing control and mutant embryos, no obvious macroscopic morphological differences were observed; however, GFP reporter expression revealed a general increase in midline YAP activity in *vgll4b* mutants (Fig. 2C). Higher-resolution analysis showed elevated GFP signal in the mid-axial notochord (quantified in Fig. S2) and in the floor plate. Strikingly, at the posterior end of the axis, YAP overactivation was most prominent in the posterior floor plate and posterior hypochord, corresponding to the region where notochord progenitors reside (Fig. 2F).

qPCR on isolated tailbuds from control and mutant embryos showed that the YAP transcriptional targets *ccn1* and *ccn2a* were upregulated in *vgll4b* mutants, whereas transcriptional readouts of other signalling pathways known to act in tailbud progenitors were unchanged (Fig. 2D). We quantified GFP intensity at 24 hpf across the posterior ends of the three midline tissues: the floor plate (Fig. 2G), hypochord (Fig. 2H) and notochord (Fig. 2I). In all three, YAP reporter activity was consistently higher in *vgll4b* mutants. Similar results were observed at later developmental stages (Fig. S2).

Together, these findings demonstrate that *vgll4b* mutants exhibit YAP overactivation throughout the posterior midline, including the notochord progenitor niche. This raises the question of whether elevated YAP activity in the mutant perturbs the rate or pattern of progenitor recruitment during notochord formation and posterior axis elongation.

### Loss of *vgll4b* expression enhances notochord progenitor addition

**Fig. 3.**
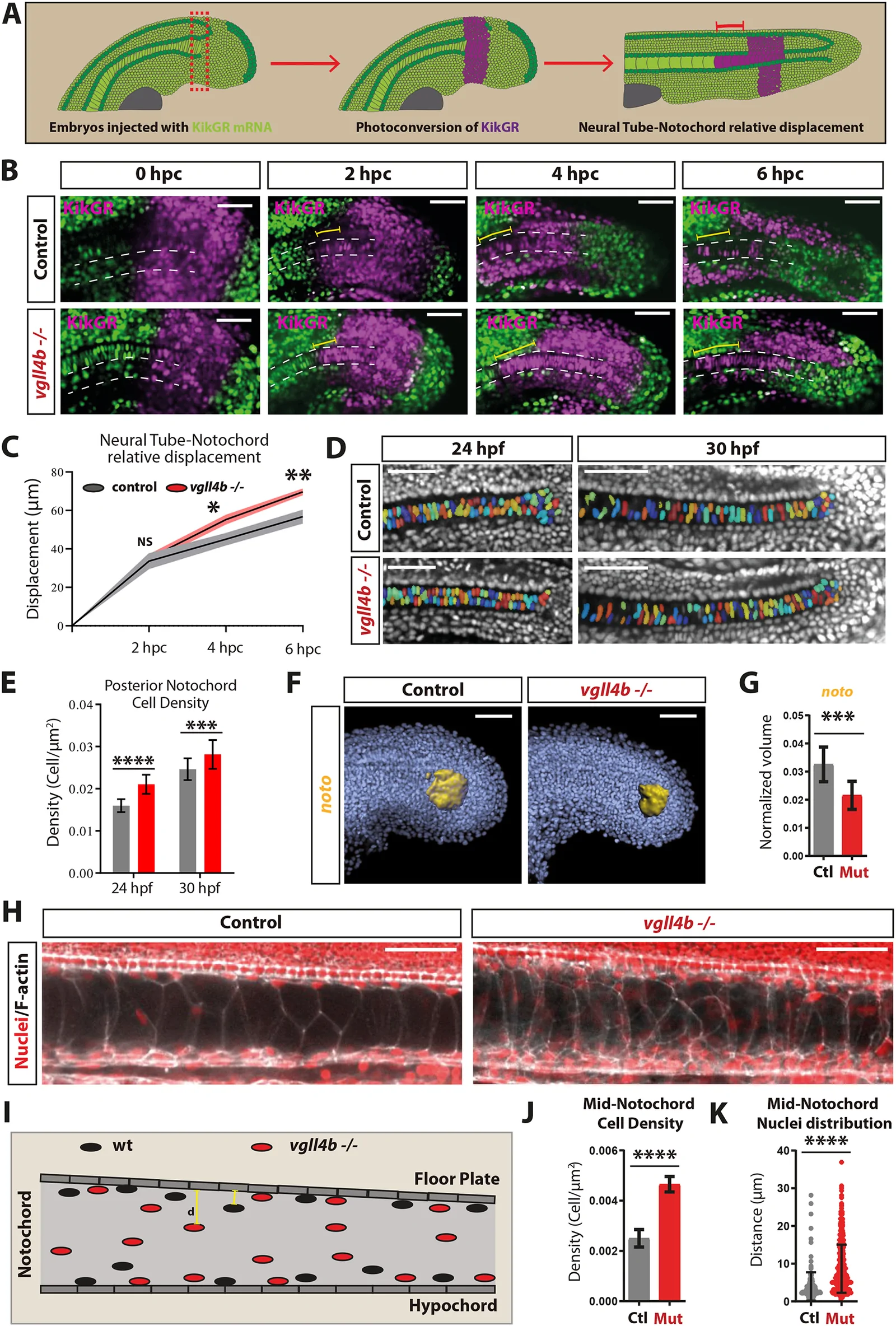
**Enhanced YAP activity in *vgll4b* mutants increases the contribution of midline progenitors to the notochord.** (A) Scheme showing the KikGR photoconversion strategy. Red dashed box highlights the photoconverted region. Red bracket indicates the displacement distance between front of the photoconverted notochord and the front of the fotoconverted floor plate. (B) Confocal images of photoconverted control and mutant embryos at different time points. Scale bars: 100 μm. 0 h post-photoconversion (hpc) corresponds to 18 hpf. Dashed lines highlight the notochord. Yellow brackets indicate displacement distance. (C) Quantification of relative notochord displacement with respect to the neural tube at 2 hpc (*t*-test; control *n*=4, mutant *n*=5; *P*=0.7288), 4 hpc (*t*-test; control *n*=5, mutant *n*=5; *P*=0.0306) and 6 hpc (*t*-test; control *n*=4, mutant *n*=5; *P*=0.0079). Solid lines represent mean values and shaded areas denote data dispersion. NS, not significant. (D) Confocal images of the posterior notochord at 24 hpf and 30 hpf in *Tg(4xGTIIC:GFP)* control and *Tg(4xGTIIC:GFP)*; *vgll4b*^−/−^ embryos, showing segmented nuclei. Scale bars: 50 μm. (E) Quantification of cell density at the posterior end of the notochord at 24 hpf (*t*-test; control *n*=12, mutant *n*=18; *P*<0.0001) and 30 hpf (*t*-test; control *n*=19, mutant *n*=23; *P*=0.0008). (F) Volumes of *noto* expression from HCR staining in control and mutant embryos at 24 hpf. Scale bars: 100 μm. (G) Quantification of normalized *noto* expression volume (*t*-test; control *n*=12, mutant *n*=10; *P*=0.0004). (H) Confocal images of the mid-axial notochord in control and mutant embryos at 30 hpf stained with DAPI and phalloidin. Scale bars: 50 μm. (I) Schematic illustrating nuclear distribution in mid-axial notochord cells of control and mutant embryos. Yellow brackets represent the distance to the nearest notochord-flanking tissue (floor plate or hypochord). (J) Quantification of mid-axial notochord cell density (*t*-test; control *n*=5, mutant *n*=5; *P*<0.0001). (K) Quantification of mid-axial notochord nuclear distribution (Mann–Whitney test; control *n*=5, mutant *n*=5; *P*<0.0001). Error bars in E,G,J,K represent s.d.

To investigate the requirement of *vgll4b* in notochord progenitor addition, we performed a lineage-tracing experiment using the photoconvertible protein Kikume Green-Red (KikGR). Embryos were injected at the one-cell stage with *KikGR* mRNA, and at the onset of tail elongation (18 hpf) a vertical band of cells was photoconverted, encompassing cells destined to form part of the spinal cord, midline structures and somitic mesoderm (Fig. 3A).

If YAP overactivation in the *vgll4b* mutant specifically affects midline progenitor behaviour rather than the general addition of cells to the axis, the relative displacement over time between the most anterior photoconverted cells in the notochord and the most anterior photoconverted cells in the spinal cord can serve as a proxy for progenitor addition to the notochord. Consistent with this hypothesis, we observed that in *vgll4b* mutants the relative displacement between the anterior fronts of photoconverted notochord and spinal cord cells increased over time (Fig. 3B,C), indicating enhanced incorporation of cells into the notochord. To corroborate this finding, we quantified cell density at the posterior end of the notochord at 24 and 30 hpf in control and *vgll4b* mutant embryos (Fig. 3D, Fig. S3H,I). At both time points, the posterior notochord of *vgll4b* mutants contained more cells than that of controls (Fig. 3E), further supporting the hypothesis of increased progenitor addition in the mutants.

To characterize the classical notochord progenitor niche, which is identified by *noto* expression, we measured the volume of the normalized *noto*-expressing domain at 24 hpf (Fig. 3F). The *vgll4b* mutants exhibited a reduction in the *noto*-expressing volume at this stage (Fig. 3G), suggesting faster depletion of this progenitor pool, consistent with the observed enhanced addition of progenitors into the notochord. To assess directly the increased incorporation of cells into the notochord, we quantified nuclear number and distribution in the mid-axial notochord at the level of the yolk extension at 30 hpf (Fig. 3H,I). *vgll4b* mutants exhibited a higher number of notochord cells (Fig. 3J) as well as altered nuclear positioning (Fig. 3K). In controls, nuclei were primarily localized near the periphery of the notochord, whereas in mutants, the increased cell number resulted in nuclei also occupying more central positions within the structure. To exclude the possibility that mutant embryos contain a higher number of notochord cells prior to tail formation, we compared notochord cell density and total notochord cell number at 17 hpf and found no differences between control and *vgll4b* mutant embryos in either measure (Fig. S3E-G).

To discard the possibility that the increased number of notochord cells in *vgll4b* mutants is due to elevated proliferation of notochord cells or their progenitors, we quantified cell divisions within the midline progenitor niche. No significant differences were observed between control and mutant embryos (Fig. S3A-D).

Taken together, these observations demonstrate that *vgll4b* mutants, in which YAP activity is elevated in midline structures, exhibit a specific increase in the addition of progenitor cells to the notochord compared with control embryos.

### Feedback between progenitor addition and vacuolation controls notochord morphogenesis

**Fig. 4.**
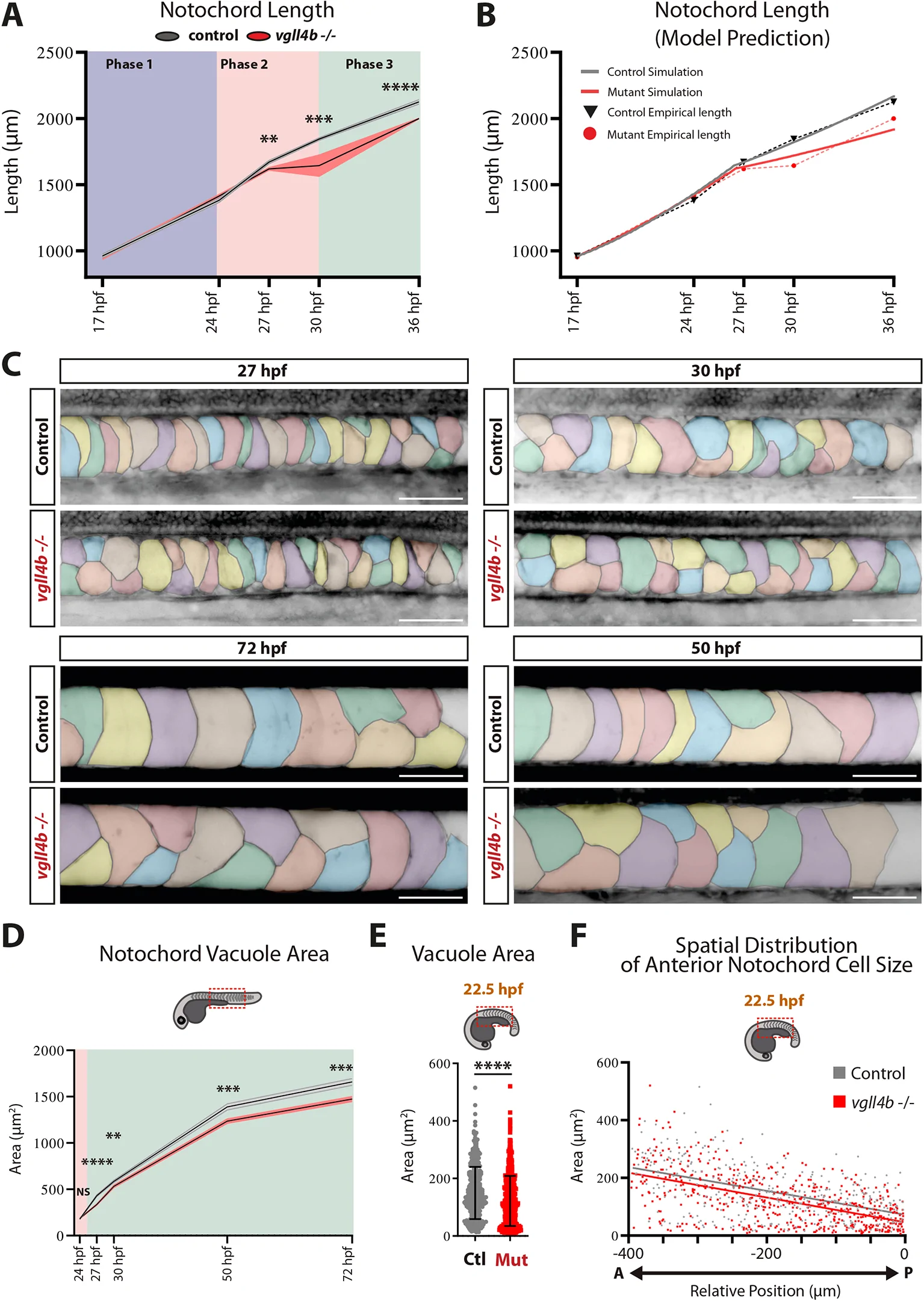
***vgll4b* mutants exhibit notochord vacuolation defects and reduced notochord length.** (A) Quantification of A–P axis length at 17 hpf (*t*-test; control *n*=12, mutant *n*=12; *P*=0.7367), 24 hpf (*t*-test; control *n*=10, mutant *n*=11; *P*=0.1232), 27 hpf (*t*-test; control *n*=20, mutant *n*=20; *P*=0.0039), 30 hpf (Mann–Whitney test; control *n*=16, mutant *n*=19; *P*<0.0001) and 36 hpf (*t*-test; control *n*=7, mutant *n*=7; *P*<0.0001). The same control measurements were used in Fig. 1F. Solid lines represent mean values and shaded areas represent data dispersion. (B) Mathematical model predictions of notochord length for control and *vgll4b* mutant embryos at different time points. The model assumes that YAP increases progenitor addition *r_p_* and simultaneously regulates the vacuolation front speed *v*_*front*_, slowing the wave of vacuolation, without affecting the vacuolation rate of each notochord cell (see Eqns 3 and 4 in the main text). The empirical measurements shown in the graph are the same as those presented in A. (C) Segmentation of notochord vacuoles in control and mutant embryos at different time points visualized with BODIPY staining. Scale bars: 50 μm. The control image at 27 hpf is the same as that shown in Fig. 1C. (D) Quantification of notochord vacuole area at 24 hpf (*t*-test; control *n*=9, mutant *n*=6; *P*=0.3993), 27 hpf (Mann–Whitney test; control *n*=8, mutant *n*=6; *P*<0.0001), 30 hpf (*t*-test; control *n*=8, mutant *n*=7; *P*=0.0083), 2 dpf (*t*-test; control *n*=9, mutant *n*=12; *P*=0.0003) and 3 dpf (*t*-test; control *n*=11, mutant *n*=12; *P*=0.0009). Solid lines represent mean values and shaded areas represent data dispersion. (E) Quantification of cell area within the anterior notochord in control and mutant embryos at 22.5 hpf (*t*-test; control *n*=8, mutant *n*=9; *P*<0.0001). Error bars represent s.d. (F) Graphical representation of the spatial distribution of anterior notochord cell size in control and mutant embryos at 22.5 hpf. The posterior boundary of the ROI used for measurement was positioned at the level of the yolk extension (0) and extended 400 μm anteriorly from this point (−400). Solid lines represent the best linear fits.

To determine the effects of enhanced posterior progenitor addition on notochord elongation rate, we quantified notochord length at multiple stages from the onset of tail elongation to 36 hpf in control and *vgll4b* mutant embryos (Fig. 4A). These measurements reveal a robust size-buffering property during the early phase of notochord extension, when posterior progenitor addition is the predominant driver of elongation. During this period, notochord length remains comparable between genotypes despite the elevated progenitor incorporation observed in *vgll4b* mutants. This buffering later fails in the subsequent phase, when vacuolation becomes the principal driver of elongation (Fig. 4A). Here, *vgll4b* mutants display a shorter notochord even though they contribute more progenitor cells to the tissue. This phenotype suggests the presence of a negative-feedback mechanism linking posterior progenitor addition to anterior vacuolation.

To investigate this hypothesis, we incorporated it into the mathematical model. We posited that YAP activity negatively regulates vacuolation-induced volume expansion while positively regulating progenitor addition. These effects were incorporated by expressing the vacuolation rate *J* and progenitor addition rate *r*_*p*_ as functions of normalized YAP levels *Y*:
(1)

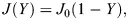

(2)

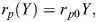
where *J*_0_ and *r*_*p*0_ denote the maximal vacuolation and progenitor addition rates, respectively. Further justification for these functional forms is provided in the Materials and Methods.

To investigate the plausibility of this hypothetical feedback between YAP and vacuolation, we analysed experimental AP profiles of notochord cell area for both wild-type and mutant embryos. As a proxy for cell area that matches our model formulation in one dimension, we have expressed this in the empirical data as nearest-neighbour distance (wild type data: Fig. S1A; *vgll4b* mutant data: Fig. S4A). Assuming scaling (see Materials and Methods), we estimated the parameters *J* and *v*_*front*_ by fitting a linear function to these profiles (see Materials and Methods). Under this hypothesis, we expected the vacuolation rate *J* to be reduced in the mutant, while the vacuolation front speed *v*_*front*_ would remain unchanged. Such a model would be consistent with a mechanism by which YAP activity in the middle region of the notochord (Fig. 2C, white arrow) inhibits vacuolation rate, in addition to its role in promoting progenitor addition. However, our estimates yielded *J*=0.66±0.07 and *v*_*front*_=70±10 for the wild type, and *J*=0.9±0.2 and *v*_*front*_=110±70 for the mutant (mean±s.d.). Furthermore, within the experimentally constrained ranges of *J* and *v*_*front*_, the model failed to reproduce the notochord elongation dynamics, in particular the phase 3 behaviour in which YAP-dependent effects on vacuolation are observed following an earlier phase where these effects are buffered by progenitor addition (Fig. S5). We therefore rejected the hypothesis that YAP modulates both the progenitor addition rate and the vacuolation rate.

We next proposed an alternative model variant in which YAP regulates the vacuolation front speed *v*_*front*_. Biologically, this corresponds to distinguishing between the propagation of a trigger or competence signal along the tissue, and the execution of vacuole growth within each cell. Our reasoning was that they need not be strictly proportional: a signalling wave could propagate at a given speed even if the downstream cellular response is slower or faster. An increase in vacuolation front speed would be consistent with an alternate mechanism by which the timing of vacuolation onset is slowed in *vgll4b* mutants, slowing the wave of vacuolation through the tissue. In this formulation, the vacuolation rate *J* is independent of YAP, and thus identical in wild-type and mutant embryos, while, as in the previous model variant, the progenitor addition rate remains higher in the mutant than in the wild type. This revised model is described by the following equations:
(3)

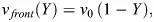

(4)

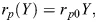
where *v*_0_ is a constant.

By adopting values of *J* and *v*_*front*_ within the experimentally derived constraints, we identified a combination that reproduced the notochord elongation dynamics in agreement with that observed experimentally (Fig. 4B). This model assumes that in *vgll4b* mutants YAP increases the rate of progenitor addition with an additional decrease in the vacuolation front speed, without affecting the vacuolation rate. To test this hypothetic alternative experimentally, we next examined vacuolation dynamics directly in *vgll4b* mutants. Notochord vacuoles were segmented, as shown in Fig. 1C, and quantified in the mid-axial notochord from 24 to 72 hpf (Fig. 4C,D). Across all time points at which notochord length is reduced in mutants (from 27 hpf onwards), *vgll4b* mutants consistently exhibited reduced vacuole area relative to controls (Fig. 4D). This reduction in vacuole area aligns with our earlier observation that mutants contain a greater number of notochord cells, which likely inhibits the ability of a cell to vacuolate. Impaired vacuolation results in a shorter notochord (Fig. 4A) and, consequently, a reduced body axis length at 2-3 days post fertilization (dpf) (Fig. S6). Shortened axial length is a well-documented outcome in zebrafish embryos with defective notochord vacuolation (Parsons et al., 2002) or fragmented vacuoles (Bagwell et al., 2020; Garcia et al., 2017; Lim et al., 2017). Notably, *vgll4b* mutants also lose the characteristic A–P scaling of notochord cell A–P length observed in controls (Fig. 1H, Fig. S4), suggesting that altered progenitor addition dynamics disrupt the intrinsic scaling properties of the system.

In contrast to 27 hpf onwards, measurements at 24 hpf showed no differences in vacuole area between genotypes (Fig. 4D), likely due to the fact that vacuolation is minimal in this region of the body axis. Given that notochord cell vacuolation initiates in the anterior region and proceeds in a posterior direction, we performed a comparable analysis of vacuole area in the anterior notochord at earlier developmental stages (22.5 hpf). This analysis similarly revealed a reduction in vacuole area in *vgll4b* mutant embryos compared with controls, consistent with the results observed in the mid-axial notochord at later stages (Fig. 4E,F). This suggests that the increased number of notochord cells in the mutant does not affect the initiation of vacuolation, but that at later stages, when vacuoles increase in size, this may constrain the extent of individual vacuole expansion. Taken together, these data suggest that the decreased anterior expansion of the notochord via vacuolation maybe compensating for increased progenitor addition, essentially maintaining overall notochord length similar to that of controls up until 24 hpf. However, as vacuolation continues to extend through to more posterior regions, notochord length begins to decrease relative to controls (Fig. 4A).

Taken together, these results demonstrate that the notochord integrates an effective feedback mechanism linking progenitor addition and anterior vacuolation in zebrafish. We propose that elevated YAP activity, by increasing progenitor density in the pre-vacuolated notochord, reduces notochord vacuolation capacity. At early stages, this explains the buffering capacity of the notochord to maintain the same rate of elongation in *vgll4b* mutants, even though progenitor addition is increased. However, upon the cessation of progenitor addition, the inability to propagate vacuolation across the full extent of the notochord results in a decrease in elongation rate.

### YAP inhibition is required for progenitor addition but not for directly inhibiting anterior vacuolation

**Fig. 5.**
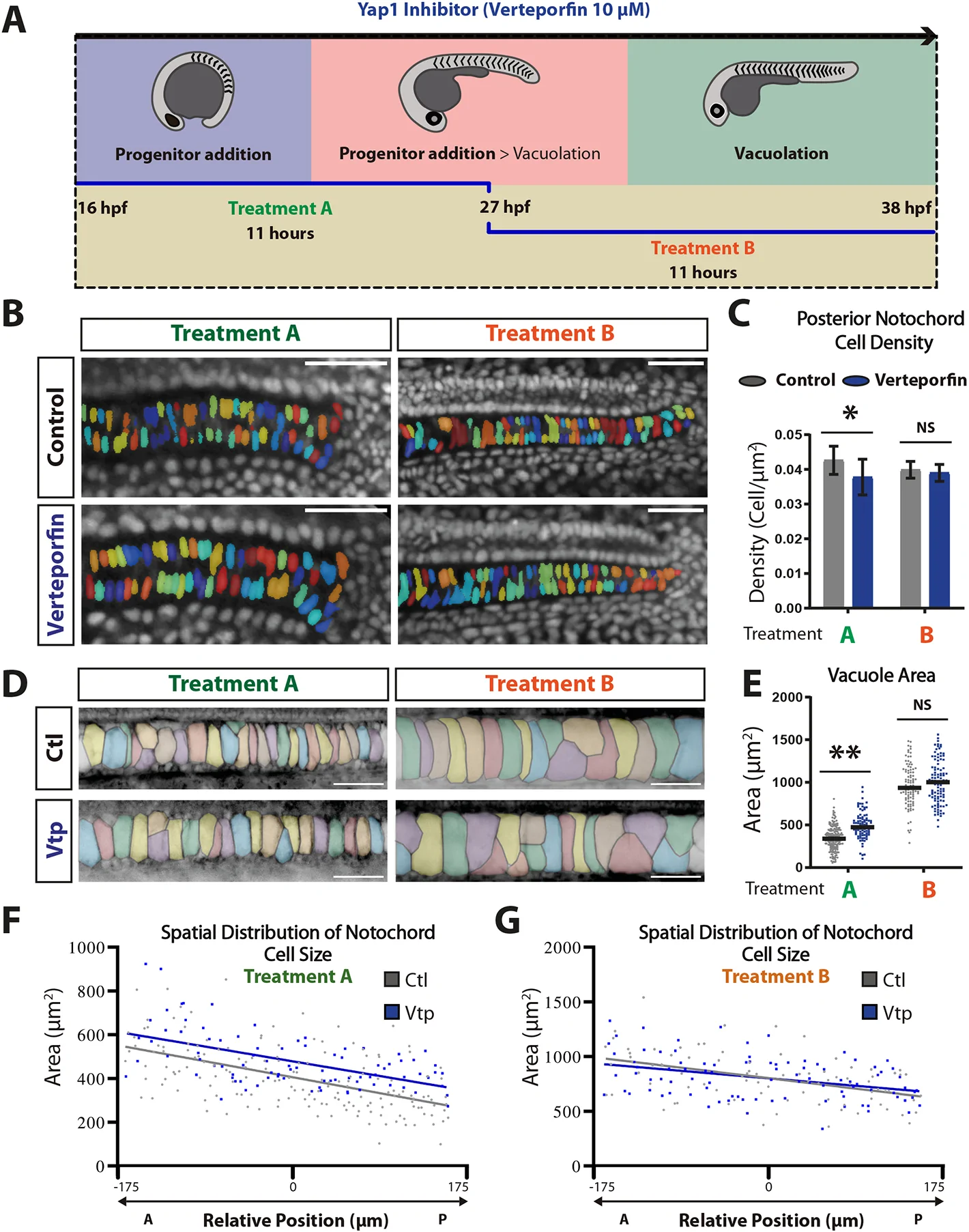
**YAP inhibition decreases notochord cell density and disrupts vacuolation.** (A) Schematic of verteporfin (YAP inhibitor) treatment strategy. Embryos were subjected to either Treatment A or Treatment B. (B) Confocal images of the posterior notochord at 27 hpf (Treatment A) and 38 hpf (Treatment B) in control and verteporfin-treated embryos, showing segmented nuclei. Scale bars: 50 μm. (C) Quantification of posterior notochord cell density: Treatment A (*t*-test; control *n*=10, treated *n*=14; *P*=0.0219); Treatment B (*t*-test; control *n*=8, treated *n*=9; *P*=0.4696). Error bars represent s.d. NS, not significant. (D) Segmentation of notochord vacuoles in control and verteporfin-treated embryos visualized with BODIPY staining. Scale bars: 50 μm. (E) Quantification of vacuole area in the central notochord: Treatment A (*t*-test; control *n*=7, treated *n*=4; *P*=0.00078); Treatment B (*t*-test; control *n*=7, treated *n*=7; *P*=0.2406). NS, not significant. Black lines represent median values. (F,G) Quantification of cell area within the notochord in control and treated embryos for Treatments A and B. The A–P axis in the graph is centred at the yolk-level extension. The ROI used for measurements spanned 175 μm on each side of this reference point, for a total length of 350 μm. Solid lines represent the best linear fits. Ctl, control; Vtp, verteporfin.

To test our proposed model, we next aimed to inhibit YAP signalling during a later phase when notochord elongation is driven only by vacuolation. If YAP plays a direct role in inhibiting vacuolation rate, this should still block anterior expansion as we still observe YAP activity in the notochord at post-somitogenesis stages (Fig. S2A,B). Conversely, if our model is correct, and the observed inhibition in *vgll4b* mutant embryos is an indirect consequence of increased progenitor addition, no adverse effects of vacuolation would be expected at these stages. To achieve temporal control over YAP inhibition, we used the well-established YAP inhibitor verteporfin to enable temporal control over YAP inhibition during distinct phases of its elongation (Fillatre et al., 2019; Ren et al., 2021; Ye et al., 2020). We designed two treatment windows (Fig. 5A). Treatment A consisted of verteporfin exposure from 16 to 27 hpf, corresponding to the phase in which posterior progenitor addition is ongoing and constitutes the primary driver of notochord and axis elongation. Treatment B consisted of drug exposure from 27 to 38 hpf, a period during which progenitor addition has ceased and vacuolation becomes the main driver of notochord extension. The effect of verteporfin in reducing YAP1 activity in the midline progenitor structures during axis elongation was confirmed (Fig. S7).

We first quantified cell density at the posterior end of the notochord using nuclear labelling (Fig. 5B). Embryos subjected to Treatment A showed a marked reduction in posterior cell density, whereas Treatment B embryos were indistinguishable from controls (Fig. 5C). This result indicates that the number of cells incorporated into posterior notochord is reduced specifically when YAP is inhibited during the progenitor-addition window.

We next quantified vacuolation under both treatment regimes. Notochord vacuoles were segmented and their area measured in the mid-axial notochord (Fig. 5D). Treatment A embryos exhibited a significant increase in vacuole area compared with controls, whereas Treatment B embryos did not show detectable differences (Fig. 5E-G). These findings are consistent with a model in which reduced progenitor addition in Treatment A results in fewer notochord cells and therefore permits greater vacuolation per cell. By contrast, YAP inhibition during the vacuolation phase (Treatment B), when progenitor input has ceased, does not influence vacuolation dynamics. Together, these results demonstrate that reduced YAP activity in midline progenitors decreases their contribution to the notochord, and later indirectly accelerates vacuolation. Conversely, the increased packing of progenitor cells in *vgll4b* mutants (Fig. 3D,E) results in a delay to anterior vacuolation, buffering the early impact of accelerated cell addition (Fig. 4A,B). Taken together, we propose a mechanism by which YAP-regulated progenitor addition indirectly inhibits anterior vacuolation, mechanically coupling progenitor addition and anterior expansion across the length of the notochord.

## DISCUSSION

In this study, we investigated the regulatory mechanisms that coordinate progenitor addition and vacuolation during zebrafish notochord morphogenesis and A–P axis elongation. Mathematical modelling and *in vivo* measurements indicated that progenitor incorporation at the posterior end contributes to tissue extension, whereas vacuolation of anterior differentiated cells generates a vacuolation front that maintains cell-size scaling along the A–P axis. YAP activity is enriched in posterior midline progenitors, coinciding with the region of active cell addition, and is negatively regulated by the inhibitor *vgll4b*. Loss of *vgll4b* results in elevated YAP activity, increased progenitor incorporation, reduced vacuolation, and, ultimately, altered notochord elongation and disrupted A–P cell-size scaling. The agreement between model predictions and experimental measurements supports the sufficiency of progenitor addition and vacuolation to explain both tissue elongation and the emergence of a linear cell-size gradient, highlighting a fundamental design principle of tissue-scale morphogenesis. These findings extend prior work on biphasic notochord elongation (Ellis et al., 2013; McLaren and Steventon, 2021) by explicitly linking progenitor dynamics with vacuolation-driven mechanical expansion.

Our data support a mechanism in which the balance between notochord progenitor addition and subsequent vacuolation collectively determines A–P axis length. We propose that the effect on vacuolation is indirect: YAP primarily regulates the rate of posterior progenitor incorporation, and the resulting increase in notochord cell number subsequently attenuates anterior volume expansion driven by vacuolation, which reduces notochord length. Effectively, this functions to couple these two processes across the length of the notochord over time, providing a long-range feedback that enables robustness in elongation to alterations in the rate of progenitor addition. This interpretation aligns with prior zebrafish studies demonstrating that disruption of notochord vacuolation, whether by pharmacological inhibition (Tang et al., 2025; Yuan et al., 2023) or by genetic mutation affecting vacuole behaviours (Bagwell et al., 2020; Coutinho et al., 2004; Ellis et al., 2013; Garcia et al., 2017; Lim et al., 2017), leads to measurable defects in axis elongation. In *vgll4b* mutants, increased progenitor incorporation initially does not alter overall notochord length. This has the potential to provide a buffering mechanism for natural variation in progenitor addition that remains to be directly tested; however, once vacuolation becomes the dominant driver of extension, mutants display shorter notochords and reduced A–P axis length.

Our observations suggest that, during the progenitor-driven phase, the notochord retains a mechanical or morphogenetic buffering capacity that transiently compensates for elevated cell addition. This buffering fails once vacuolation dominates, revealing a feedback interaction between posterior progenitor incorporation and anterior vacuole expansion. Comparable buffering phenomena have been described in zebrafish using mechanical perturbations: robot-assisted micromanipulation has shown that axial tissues elongation resist perturbations through coordinated cell rearrangements and tension modulation (Özelçi et al., 2022). In addition, proportional regulation of posterior body elongation has been linked to spinal cord-mediated coordination of notochord and mesodermal tissue growth upon targeted ablation of spinal cord progenitors (Saunders et al., 2025). Together, these results build on prior analyses of tissue mechanics during axis elongation and highlight the notochord as a dynamic structure in which cellular composition, mechanical properties and morphogenetic scaling converge to shape vertebrate body axes (Adams et al., 1990; Mongera et al., 2018).

Interestingly, Vgll4 has been implicated in body-size regulation in the fish *Scatophagus argus* (Yang et al., 2020), and mouse mutants exhibit a shortened body axis (Feng et al., 2019; Sheldon et al., 2022; Suo et al., 2020; Yu et al., 2019). In zebrafish, the maternal contribution of *vgll4a* and the zygotic contribution of *vgll4b* have been shown to be required for establishing the correct A–P axis length (Camacho-Macorra et al., 2024). Whereas *vgll4a* regulates YAP activity during epiboly to fine-tune axis length, the mechanism underlying the zygotic role of *vgll4b* remained unclear. Although VGLL4 is a well-established YAP inhibitor, recent studies indicate that it can also regulate gene expression independently of the VGLL4–YAP–TEAD axis in stem cell systems (Quan et al., 2023; Wang et al., 2023), in mouse (Suo et al., 2025; Teng et al., 2010; Zhang et al., 2026) and in zebrafish (Fillatre et al., 2019; Lardennois et al., 2026; Wang et al., 2020; Xue et al., 2019). In our dataset, however, *vgll4b* appears to function predominantly through YAP regulation. Tailbud-specific transcriptional profiling of *vgll4b* mutants revealed upregulation of canonical YAP targets (*ccn1*, *ccn2a*) without significant changes in other pathways known to influence posterior progenitors. Moreover, pharmacological YAP inhibition produced phenotypes opposite to those of *vgll4b* mutants. These findings indicate that the principal role of *vgll4b* in this context is to constrain YAP activity. One caveat of this experiment is the known function for verteporfin in lysosomal disruption (Gavini et al., 2019); however, we do observe a dysregulation of YAP activity within posterior midline structures (Fig. S7) and the opposing phenotypes observed with *vgl4b* mutants support a specific role of YAP activity in this scenario. This spatially restricted antagonism ensures that YAP promotes progenitor maintenance and incorporation at the posterior end while preventing excessive accumulation that would compromise vacuolation.

Although our study establishes a mechanistic framework for YAP-mediated coordination of progenitor addition and vacuolation, several limitations remain. Our model represents the notochord as a one-dimensional chain of uniform cells, which may oversimplify the three-dimensional architecture as well as its mechanical properties. Furthermore, the downstream YAP effectors in midline progenitors and the mechanisms governing their incorporation into the notochord remain to be identified. While YAP is a prominent promoter of cell proliferation in both physiological and cancer contexts (Luo et al., 2023; Pocaterra et al., 2020), the extremely rapid pace of zebrafish development, coupled with low proliferation rates in the tailbud, indicates that axis elongation is driven primarily by cell movements and rearrangements rather than cell division (Attardi et al., 2018; Bouldin et al., 2014; Kanki and Ho, 1997). This is consistent with observations that pharmacological inhibition of proliferation does not prevent A–P axis extension (Stooke-Vaughan et al., 2025). Notably, VGLL4 antagonism of YAP activity has been shown to reduce cell migration in cultured cells (Mickle et al., 2021; Sun et al., 2022) and *in vivo* models (Zhang et al., 2014, 2017). We therefore hypothesize that elevated YAP activity in *vgll4b* mutant progenitors increases their rate of incorporation into the notochord rather than enhancing their proliferation. This hypothesis is supported by our observation of no differences in cell proliferation between mutants and controls during the developmental window of axis elongation analysed in this study.

In summary, our study identifies YAP as a key regulator of progenitor addition and vacuolation during zebrafish notochord morphogenesis. By integrating experimental data with computational modelling, we demonstrate that a feedback mechanism balances posterior progenitor incorporation with anterior vacuole expansion to maintain notochord elongation and A–P scaling. Understanding how molecular signals interface with tissue mechanics to ensure robust scaling has broad implications for congenital axial malformations and may inform strategies for engineering notochordal or axial tissues (Nájera et al., 2026 preprint). More broadly, the interplay between progenitor dynamics and volumetric expansion may represent a general principle through which embryos coordinate multiple morphogenetic processes to achieve robust patterning and size control.

## MATERIALS AND METHODS

### One-dimensional model of zebrafish notochord elongation

We modelled the notochord as a one-dimensional tissue composed of cells. Each cell is represented as a spring–mass element: a spring with elastic constant *k* anchored at one end and connected to a mass *m* at the other. The tissue consists of a sequence of these spring–mass units arranged along the *x*-axis, corresponding to the anterior–posterior (AP) axis. The first spring is attached to a fixed anchor point (a ‘wall’) at the most anterior end of the notochord. New cells are added posteriorly by attaching the anchor point of the new cell to the mass of the current last cell.

All cells obey Hooke's law with the same elastic constant *k*, the same mass *m* (set arbitrarily to 1), and a target length *S*_*i*_, defined as the spring's rest length (see Fig. 1B for a schematic). The position of mass *i* (and thus of cell *i*) changes according to the net force exerted by its two neighbouring springs, 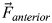 and 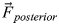. A viscous drag force, appropriate for the low–Reynolds number regime, opposes motion. We assumed that notochord cells are in an overdamped regime where the inertial term is negligible compared with the viscous forces. Applying Newton's second law for the mass *i* gives:
(5)

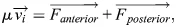

(6)

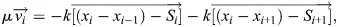
where 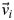 is the velocity of cell *i*, *x*_*i*_ is its position, *S*_*i*_ is its target size and *μ* is the drag coefficient. On the right-hand side of Eqn 6, we use a simplified notation to represent the forces acting on each mass *i* within a cell due to the two adjacent springs. Since the cells are arranged along the anterior–posterior axis, we represent the model in one dimension and therefore drop the vector notation from this point onwards.

We assumed the presence of a vacuolation front that starts at time 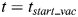 at *x*=0 (the anterior anchor point) and moves posteriorly at constant speed *v*_*front*_. A cell becomes vacuolated when its position lies anterior to the front. Upon vacuolation, the target size *S*_*i*_ increases according to:
(7)

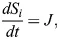
where *J* is a constant representing the vacuolation-induced rate of size increase. The solution is:
(8)

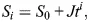
where *S*_0_ is the initial cell size (identical for all cells), and *t*^*i*^=*t*−*t*^*vacuolated*^ is the time elapsed since cell *i* became vacuolated.

Progenitor addition is implemented by inserting new cells at the posterior end at regular intervals *t*_*add*_, related to the progenitor addition rate *r*_*p*_ by *r*_*p*_=1/*t*_*add*_. Progenitor addition stops after a time *T*_*p*_ from the start of the simulation. Each time a progenitor cell is added, a small anteriorly directed force is applied to its mass, effectively compressing the tissue. In the initial condition of the simulation, we assumed *N*_0_ cells of size *S*_0_ distributed between *x*=0 and *x*=*L*_0_, representing the anterior and posterior borders of the notochord, respectively. This initial configuration corresponds to the notochord at 16 hpf, with the vacuolation wave initiating at *x*=0 while a progenitor is added at *x*=*L*_0_. All model parameters are defined in Table S3.

### Condition for cell-size scaling in the notochord

In this section, we show that during the second phase, after progenitor addition ceases, the model predicts a linearly decreasing cell-size profile along the A–P axis. We refer to this phenotype as ‘scaling’, because the shape of the distribution is conserved as the tissue grows.

This behaviour arises because vacuolated cells are pushed posteriorly by the expansion of anterior cells. Specifically, when a cell *i* at position *x*_*i*_ becomes vacuolated, its posterior displacement is driven by the *N*(*x*_*i*_, *t*) anterior cells that are also enlarging. Although the position of cell *i* could in principle be determined from the positions of all anterior cells, its velocity can be expressed more directly: it equals the cumulative size-increase rate of all anterior cells.

We assume that all cells are modelled as rigid springs (with high spring constant *k*), so that their actual length closely follows their target length *S*_*i*_; hence, we refer to cell length and target length interchangeably. Since there are *N*(*x*_*i*_, *t*) anterior cells and each grows at rate *J*, the posterior velocity of cell *i* is given by *v*_*i*_=*N*(*x*_*i*_, *t*)*J*.

From the perspective of a reference frame centred on cell *i*, the vacuolation front appears to move with an effective speed:
(9)

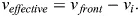
In this reference frame, the vacuolation wave front therefore advances at a constant speed. From the time the cell became vacuolated, 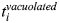, up to the current absolute time *t*, the front has travelled a distance Δ*x*. This distance corresponds to the separation between the front and the cell:
(10)

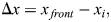
where *x*_*i*_ is the position of cell *i*. Because the vacuolation wave front moves at constant speed in this cell-centred frame, we can also express this distance as:
(11)

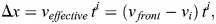
where 
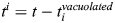
 is the time elapsed since the front passed cell *i*.

Combining the two expressions for Δ*x* gives:
(12)

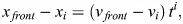
and therefore:
(13)

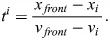
Finally, since the vacuolation wave front advances at constant speed, its position is simply:
(14)

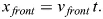
Hence, we obtain:
(15)

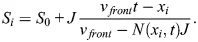
We can replace the index *i* by a general position *x*, yielding:
(16)

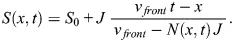
If *v*_*front*_≫*N*(*x*, *t*) *J*, this expression simplifies to:
(17)

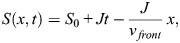
which is a straight line in *x* with constant slope and an intercept that increases linearly in time:
(18)

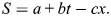


Where *a*=*S*_0_, *b*=*J* and 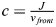. We refer to this as scaling when the spatial distribution of cell sizes at different times forms straight lines with identical slopes but different intercepts.

To study the conditions under which this linear profile of cell sizes emerges, we bounded the term *N*(*x*, *t*) in Eqn 14. Because new cells are added exclusively at the posterior tip, *N*(*x*, *t*) must lie between the initial number of cells and the maximum number that can accumulate during the period of progenitor addition. Thus, *N*(*x*, *t*) is bounded as follows:
(19)

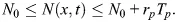
The condition *v*_*front*_≫*N*(*x*, *t*) *J* is thus guaranteed for all *x* and *t* provided that:
(20)

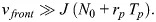
We define the dimensionless parameter as:
(21)

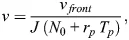
which represents the ratio between the speed of the vacuolation front and the maximal speed at which the notochord could expand.

The condition for obtaining the linear profile *S*=*a*+*bt*−*cx* therefore simply becomes *v*≫1.

When *v*≫1, we can ensure that the spatial distribution of cell sizes along the notochord is a straight line with a constant slope and a time-increasing intercept.

### Constraining the model parameter space

Eqn 17 describes a linear function for the spatiotemporal distribution of cell notochord sizes under the hypothesis that *v*_*front*_≫*N*(*x*, *t*) *J*. If we look at this equation at a fixed time, we can realize that the slope of it is 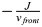. Alternatively, if we look at it at a fixed position, the slope is now *J*. If we measure these two slopes, we will end up with two equations with two unknowns (*J* and *v*_*front*_), which we can solve for.

We fitted a line at the A–P profiles of nearest-neighbour cell distances centre to centre within the notochord at 27, 30 and 33 hpf for the wild type (Fig. S1A) and mutant (Fig. S4A). To calculate the slope of Eqn 17 at fixed time, we estimated the average and standard deviation of the slopes of the fitted lines at 27, 30 and 33 hpf for the wild type and we repeated this procedure for the mutant. To estimate the slope of the wild type at fixed position, we followed this procedure. We selected a position and calculated the cell size of the wild type at that position at 27, 30 and 33 hpf from their corresponding fitted lines. We took those three values and fitted a line to A–P profiles of nearest-neighbour cell distances centre to centre within the notochord as a function of time and we obtained the slope of that line. Then, we repeated this procedure with other positions within the measured domain spaced by 0.001 μm and calculated the slope again. Finally, we calculated the mean and standard deviation among all those slopes. We repeated this procedure with the mutant. With the two slopes estimated, we solved the system of equations to calculate the values of *J* and *v*_*front*_ and used propagation of errors to calculate the standard deviation of *v*_*front*_.

### Animal husbandry

Adult zebrafish were maintained and all regulated procedures were conducted in accordance with the Animals (Scientific Procedures) Act 1986, as amended in 2012, following approval by the Animal Welfare and Ethical Review Body (AWERB) at the University of Cambridge. Embryos were kept in standard E3 medium throughout all experimental procedures. Developmental staging followed established criteria (Kimmel et al., 1995). To suppress spontaneous muscle contractions, embryos were incubated in tricaine (ethyl 3-aminobenzoate; Sigma-Aldrich, A5040) at a final concentration of 0.16 g/ml in E3 medium. The zebrafish lines used were *Tg(4xGTIIC:GFP)* (Miesfeld and Link, 2014) and *vgll4b*-P156Rfs5 (Camacho-Macorra et al., 2024).

### Pharmacological treatments

Live imaging was performed using the vital dye BODIPY TR methyl ester (MED; Invitrogen), following a standard protocol (Ellis et al., 2013). Embryos were incubated in 2% MED for 45 min, rinsed several times in E3 medium, mounted, and imaged immediately.

For verteporfin treatment, dechorionated embryos were maintained at 28°C in E3 medium containing either DMSO (vehicle control) or verteporfin (Tocris, 5305) dissolved in DMSO. Verteporfin was applied at a final concentration of 10 μM.

### Fixation and staining

Embryos were fixed in 4% paraformaldehyde for 24 h at 4°C. After fixation, samples were washed in PBS without calcium and magnesium [PBS(−/−)] containing 0.05% Tween-20 (PBST). Nuclei were stained with DAPI (1:500), and filamentous actin was labelled with Alexa Fluor 647-conjugated phalloidin (1:1000) in PBST supplemented with 0.1% DMSO. Staining was carried out for at least 24 h at 4°C.

*In situ* hybridization (ISH) probes (Fig. S1) specific for each *vgll4b* paralogue (*vgll4a*, *vgll4b* and *vgll4l*) were generated by RT-PCR using cDNA from different developmental stages and gene-specific primers (Table S1) with the Expand High Fidelity PCR System (Roche). Amplicons were cloned using the StrataClone PCR Cloning Kit (Agilent). Digoxigenin-UTP-labelled antisense riboprobes were synthesized *in vitro* using the DIG RNA Labeling Mix (Roche). Probes were precipitated overnight in LiCl at −20°C, pelleted by centrifugation at 12,000 ***g*** for 30 min at 4°C, washed with 70% ethanol, centrifuged again at 12,000 ***g*** for 15 min, dried at 65°C, and resuspended in a 1:1 mixture of RNase-free water and formamide (ITW reagents). ISH was performed using standard procedures (Camacho-Macorra et al., 2021) and visualized with NBT/BCIP.

Fluorescence ISH was performed using the hybridization chain reaction method (Choi et al., 2018) and was followed by immunohistochemistry (Sorrells et al., 2013). Primary antibodies were: chicken anti-GFP (1:200; Abcam, Ab13970; RRID: AB_300798), rabbit monoclonal anti-YAP (1:1000; Cell Signaling Technology, 8418) and mouse anti-Histone H3 (1:500; Abcam, mAbcam 10799). Goat-derived secondary antibodies were applied at a 1:1000 dilution: Alexa Fluor 488 anti-chicken (Thermo Fisher Scientific, A-11039; RRID: AB_2534096), Alexa Fluor 633 anti-rabbit (Invitrogen, A21071) and Alexa Fluor 488 anti-mouse (Thermo Fisher Scientific, A32723; RRID: AB_2633275). Nuclear counterstaining was carried out with DAPI (1:500; Sigma Aldrich, D8417-1MG) as the final step of the procedure.

### Confocal microscopy

For live imaging, embryos were mounted as described (Hirsinger and Steventon, 2017). For fixed samples, embryos were fully dissected away from the yolk, and the head was removed to allow the body to lie flat along the lateral axis. Embryos were mounted in 80% glycerol between two 1H coverslips adhered with double-sided tape. Imaging was performed on an inverted Zeiss LSM700 confocal microscope. Whole embryos were imaged in tiled sections using a 20× objective with 2× line averaging. Laser intensities, gain, and pixel resolution were kept constant across experiments.

### Photolabelling

Photolabelling was performed using the photoconvertible protein Kikume (Hatta et al., 2006). The plasmid nls-Kikume (gift from Ben Martin, SUNY Stony Brook, USA) was used to synthesize mRNA with the mMessage mMachine kit (Thermo Fisher Scientific, AM1344). Embryos were microinjected prior to the first cell division with a 100 μm diameter droplet containing 300 ng/μl Kikume mRNA. Photoconversion was performed using a 405 nm laser on a Zeiss LSM700 confocal microscope. A 30-s exposure was applied to a rectangular region of interest (ROI) positioned at the posterior end of the notochord. Upon exposure, the Kikume fluorophore underwent a shift from green fluorescence (KikG) to red fluorescence (KikR), indicating successful photoconversion.

### Visualization and registration

Image analysis was performed using Fiji (Schindelin et al., 2012). Two-dimensional length measurements were obtained using the ‘segmented line’ ROI tool in Fiji. Individual image tiles were acquired and subsequently stitched using a custom script that performed successive rounds of pairwise stitching via the Fiji ‘Stitching’ plugin (Preibisch et al., 2009). When necessary, images were rotated using the ‘TransformJ’ plugin (Meijering et al., 2001) to align anatomical structures.

### Measurements and quantifications

Tissue lengths were measured using the ‘segmented line’ tool in Fiji. Notochord length was defined from the anterior boundary of the first somite to the posterior end of the notochord.

Notochord vacuole area was quantified from the central plane of *z*-stacks. Individual vacuoles in BODIPY-stained embryos were manually segmented, and their areas measured. To determine the position of each cell along the axis, a 400 μm rectangular ROI centred on the yolk extension was used. Each vacuole within the ROI was segmented, polygon coordinates were extracted, and area calculated.

Cell density at the posterior notochord was quantified by selecting the central plane of the *z*-stack and applying a 100 μm ROI positioned using the floor plate and hypochord as landmarks. DAPI-labelled nuclei were counted and normalized to the ROI area. For notochord cell quantification at 17 hpf (Fig. S3E), an ROI encompassing the notochord between the anterior boundary of the first somite and the posterior boundary of the last somite was defined. Nuclei within this ROI were subsequently segmented using Cellpose software.

To assess nuclear distribution at the mid-notochord in control and mutant embryos, a 400 μm square ROI centred on the yolk extension was applied. The distance from each DAPI-labelled nucleus to the nearest boundary (floor plate or hypochord) was measured using the ‘segmented line’ tool in Fiji.

For GFP intensity quantification (Fig. 2F,G; Fig. S5A), a 400 μm ROI centred on the yolk extension in the central plane of the notochord was used. GFP intensity was normalized to phalloidin intensity within the same ROI. To quantify GFP along the posterior floor plate, hypochord or notochord, a straight line was drawn from the posterior notochord anteriorly, and pixel intensities extracted. GFP values were normalized to phalloidin and corrected by subtracting background signal measured from a separate region where no GFP was expressed.

Volume quantification of expression domains was performed using Imaris. Surfaces of interest were segmented from confocal stacks, and volumes calculated. Each volume was normalized to the total volume of the DAPI-labelled tailbud, defined using the posterior boundary of the last somite.

For measurements of embryo length at 2 and 3 dpf, embryos were anaesthetized in tricaine, oriented vertically, and imaged at 13× magnification using a Leica MZ10F stereomicroscope equipped with a Leica 295 camera.

For the quantification of PH3-positive cells (Fig. S3A), a circular ROI encompassing the *noto* expression domain obtained in Fig. 3 was centred on the posterior end of the notochord. In addition, rectangular ROIs were manually defined to include the five most posterior cells of both the hypochord and floor plate. PH3-positive cells within these regions were then identified and quantified.

To analyse the spatial distribution of posterior notochord cells (Figs S3 and S7), a 100 μm ROI was defined extending anteriorly from the posterior tip of the notochord. Individual notochord cells within this region were manually segmented, and the coordinates of each cell were extracted. For each position along the anteroposterior (*x*) axis, the total number of cells was normalized to the number of embryos analysed. The resulting values were then plotted to represent the average number of cells present at each position along the *x*-axis.

### qPCR experiments

Total RNA was extracted with TRIzol (Thermo Fisher Scientific) and resuspended in nuclease-free water. RNA concentration was quantified using the Qubit RNA High Sensitivity Kit (Thermo Fisher Scientific, Q32852). First-strand cDNA was synthesized using the SuperScript III First-Strand Synthesis System (Thermo Fisher Scientific, 18080051). For primer standardization, control cDNA was diluted 1:10, 1:20, 1:30 and 1:40. Ct values for each gene-specific primer pair were compared with those of the housekeeping gene *cyyr1* (Casadei et al., 2011). Standardized primers were then used to quantify gene expression across treatment groups. All experimental qPCRs were performed using cDNA diluted 1:40. Primer sequences are listed in Table S2.

### Statistical analysis

All graphs and statistical analyses were generated using GraphPad Prism 7. Two-group comparisons were performed using unpaired *t*-tests for data with parametric distributions or Mann–Whitney tests for data with non-parametric distributions. Statistical significance was reported using the following thresholds: *P<0.05, **P<0.01, ***P<0.001 and ****P<0.0001.

## AI tools declaration

During the preparation of this manuscript, the artificial intelligence tool Gemini (Google) was used exclusively for language refinement and editing of specific sections of the English text. The authors reviewed and edited the output as necessary and take full responsibility for the content, scientific accuracy, and integrity of the published work.

## Supplementary Material



10.1242/develop.205951_sup1 Supplementary information
