# Supplementary material for: Coupling Vgll4b/Yap-regulated posterior cell addition with anterior vacuolation enables robust notochord elongation
Source: Development. 2026 Sep 14;153(17):dev205951. doi: 10.1242/dev.205951 (PMC13615686; doi:10.1242/dev.205951)
Supplement: Supplementary file 1 [file develop-153-205951-s1.pdf]

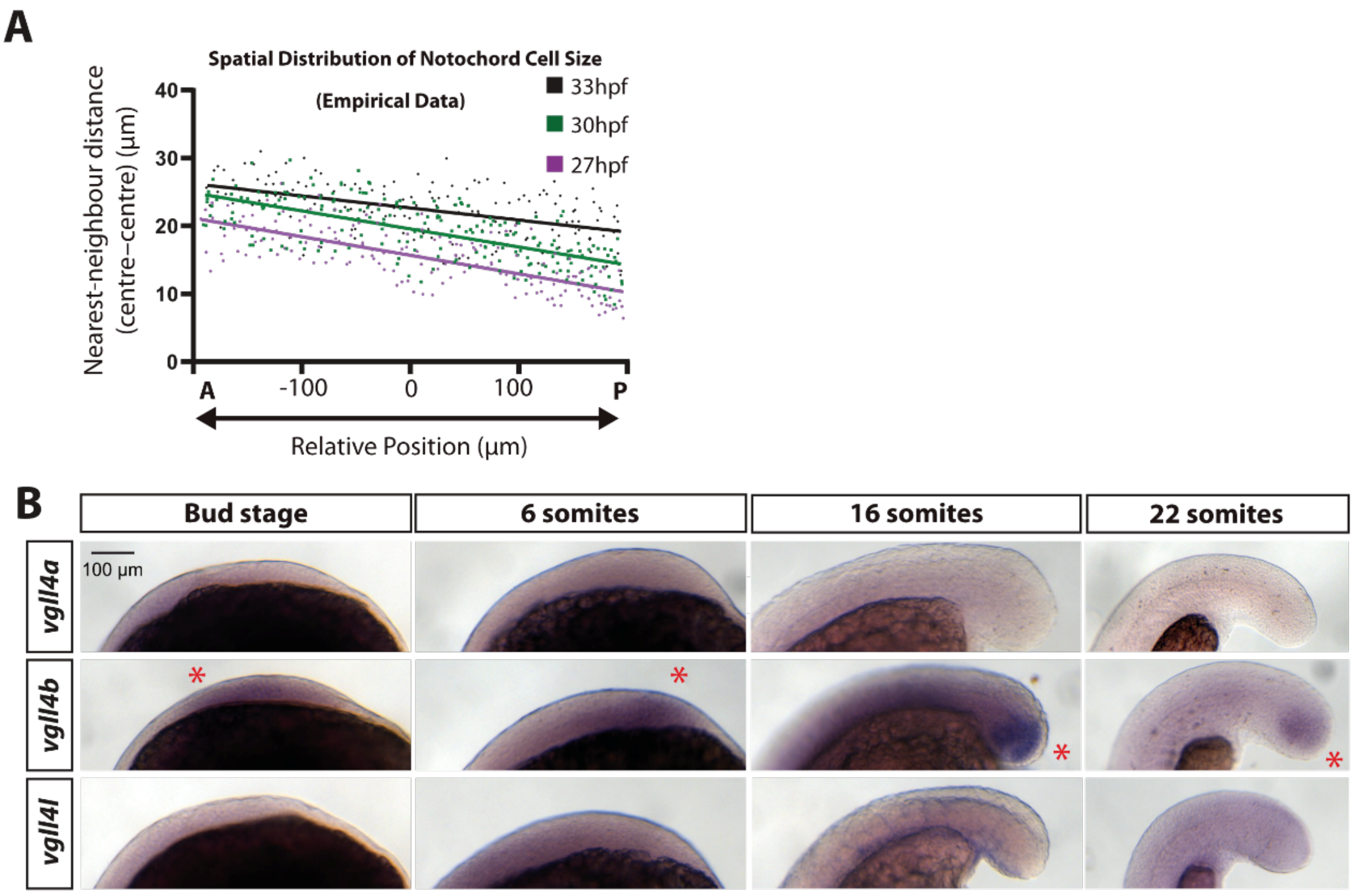

Fig. S1. Expression patterns of zebrafish *vgll4* paralogues in the posterior embryonic axis during A–P elongation.

A. Quantification of nearest-neighbour distance within the notochord at 27 hpf, 30 hpf, and 33 hpf. The A–P axis in the graph is centred at the yolk-level extension. The region of interest (ROI) used for measurements spanned 200 µm on each side of this reference point, for a total length of 400 µm. **B.** ISH staining of VGLL4 zebrafish paralogues *vgll4a, vgll4b*, and *vgll4l* in the tail at diSerent developmental stages (scale bar: 100 µm). Red asterisk points *vgll4b* expression.

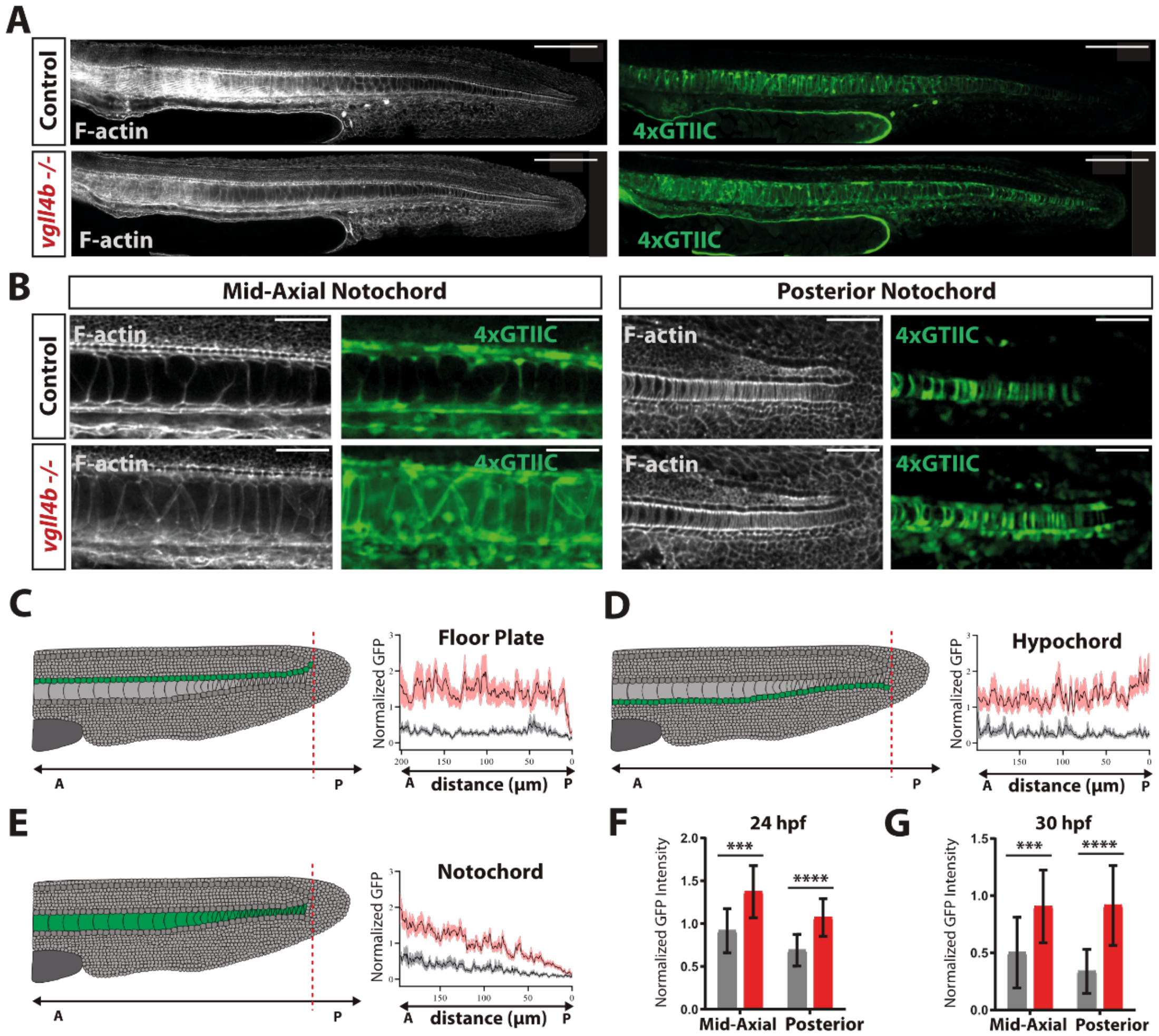

Fig. S2. Increased YAP activity in the midline structures of *vgll4b* mutants.

A. Confocal images of 30 hpf *Tg(4xGTIIC:GFP)* control and *Tg(4xGTIIC:GFP)*; *vgll4b-/-* embryos (scale bar: 100 µm). **B**. Confocal images of the mid-axial and posterior notochord in control and mutant embryos at 30 hpf (scale bar: 50 µm). **C**, **D**, **E**. Quantification of normalized GFP intensity across the floor plate, notochord, and hypochord at 30 hpf, showing sustained YAP overactivation in mutants (control n = 17, mutant n = 23), the control group is represented by the gray line, and mutants by the red line. **F**. Quantification of normalized GFP intensity in the mid-axial and posterior notochord at 24 hpf: mid-axial (t-test; control n = 10, mutant n = 18; p = 0.0005), posterior (t-test; control n = 13, mutant n = 17; p < 0.0001). **G**. Quantification of normalized GFP intensity in the mid-axial and posterior notochord at 30 hpf: mid-axial (t-test; control n = 16, mutant n = 21; p = 0.0005), posterior (Mann–Whitney test; control n = 18, mutant n = 23; p < 0.0001).

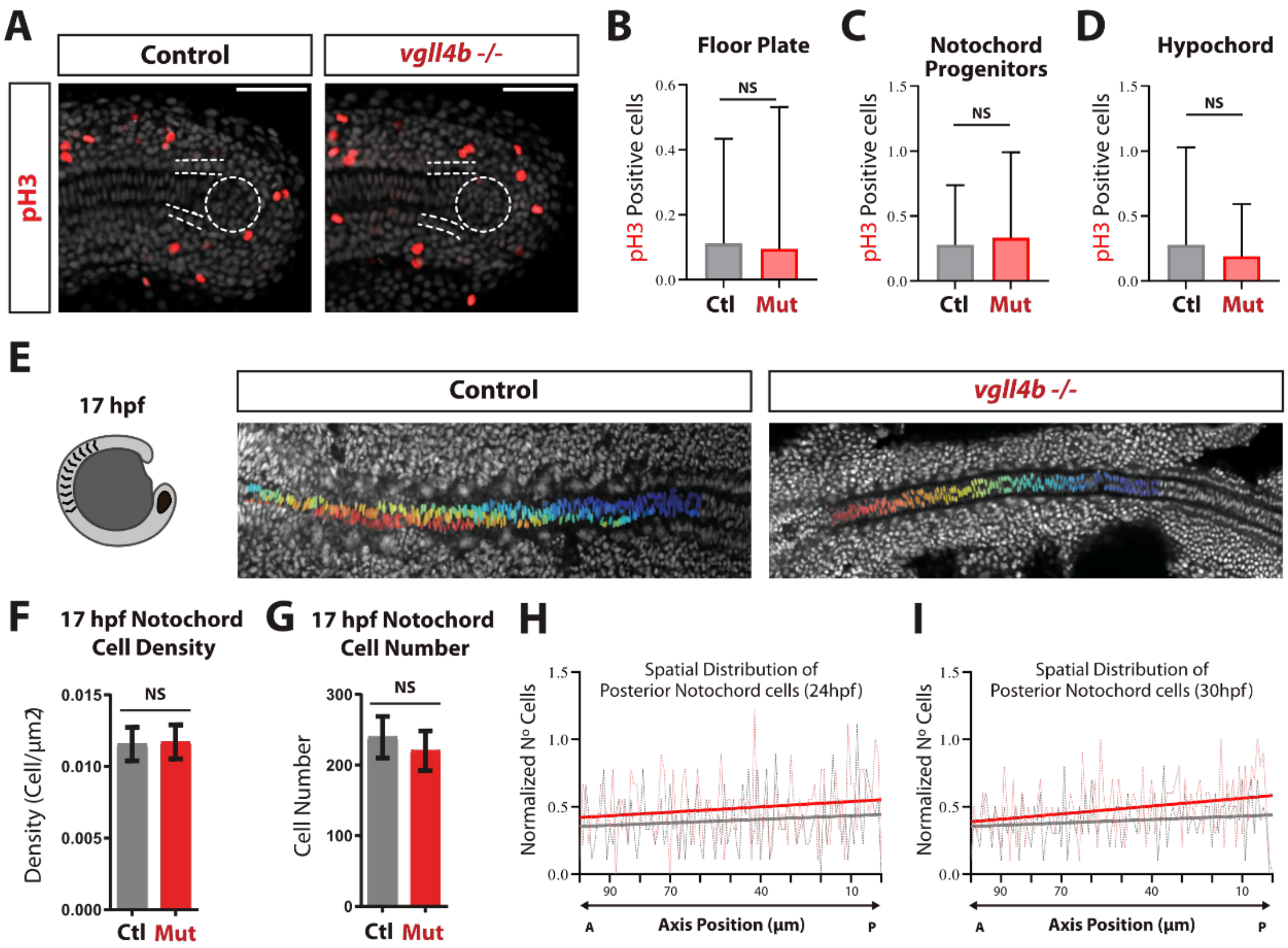

Fig. S3. ***vgll4b*** **deficiency does not alter cell proliferation or initial cell density in the embryonic notochord.**

A Confocal images of the tailbud of control and mutant embryos at 22 hpf with pH3 immunostaining (scale bar: 50 µm). Dotted white lines highlight the posterior end of the floor plate and hypochord, while the dotted white circle marks the classical notochord progenitor niche. **B**. Quantification of pH3 positive cells at the posterior end of the Floor plate (t-test; control n = 18, mutant n = 21; p = 0.8995). **C**. Quantification of pH3 positive cells in the notochord progenitor niche (t-test; control n = 18, mutant n = 21; p = 0.7657). **D**. Quantification of pH3 positive cells at the posterior end of the Hypochord (Mann-Whitney test; control n = 18, mutant n = 21; p = 0.9999). **E**. Confocal images of the notochord in control and mutant embryos at 17 hpf, prior to tail formation. Notochord nuclei were segmented for quantitative analysis. **F**. Quantification of notochord cell density at 17 hpf (t-test; control n = 10, mutant n = 7; p = 0.7926). **G**. Quantification of notochord cell number at 17 hpf (t-test; control n = 10, mutant n = 7; p = 0.1981). **H, I**. Graphical representation of the normalized number of notochord nuclei across the posterior axis in control and mutant embryos at 24 hpf (control: n = 9, 362 cells; mutant: n = 9, 442 cells) and 30 hpf (control: n = 10, 401 cells; mutant: n = 10, 491 cells). The region of interest spans 100 µm, beginning at the posterior end of the notochord, which corresponds to position 0 on the x-axis of the graph. The normalized number of notochord cells counted at each position along the axis is represented on the y-axis.

Development • Supplementary information

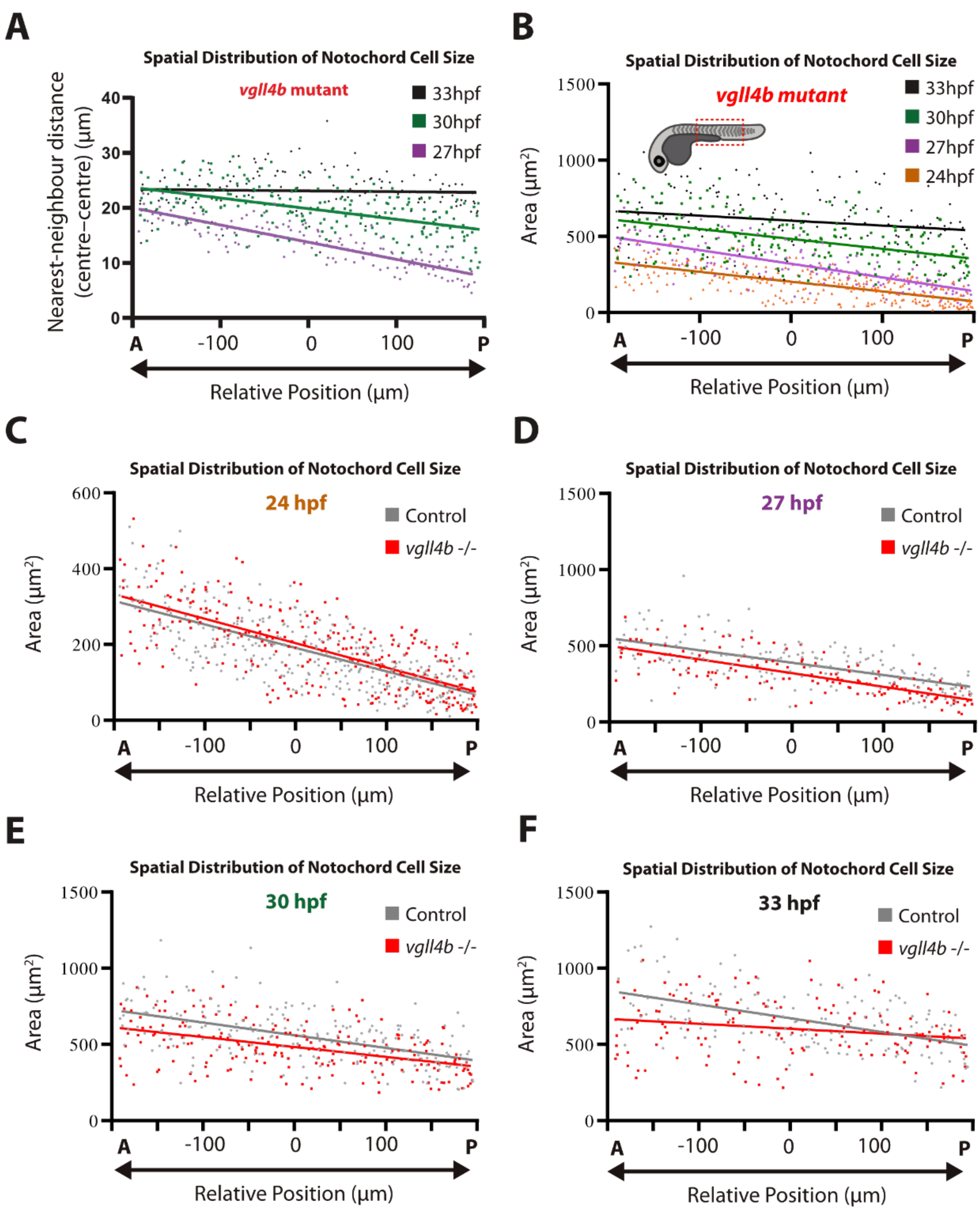

Fig. S4. Altered Spatial Distribution of Notochord Cell area in *vgll4b* Mutant Embryos

A. Quantification of AP profiles of nearest-neighbour distance within the notochord at 27 hpf, 30 hpf, and 33 hpf in *vgll4b* mutant embryos **B**. Quantification of AP profiles of cell area within the notochord at 24 hpf, 27 hpf, 30 hpf, and 33 hpf in *vgll4b* mutant embryos. **C-F**. Quantification of cell area within the notochord in control and mutant embryos at 24, 27, 30, and 33 hpf. The A–P axis in the graph is centered at the yolk-level extension. The region of interest (ROI) used for measurements spanned 200 μm on each side of this reference point, for a total length of 400 μm.

Development • Supplementary information

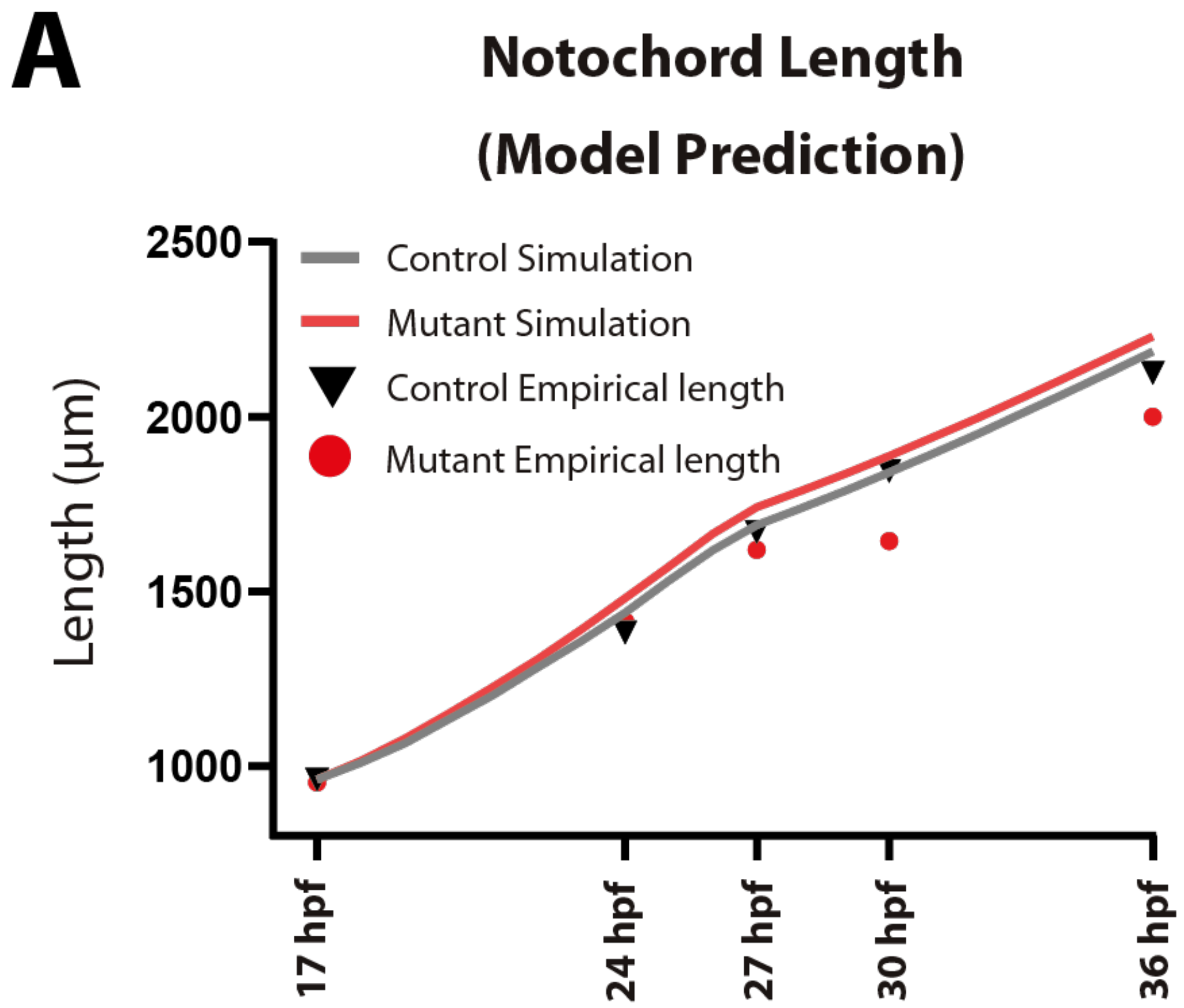

Fig. S5. Predictions of mathematical model of notochord length

Mathematical model predictions of notochord length with vacuolation rate J and progenitor addition rate r_p as functions of normalized YAP levels Y for control and *vgll4b* mutant embryos at different time points (see Eqs. 1 and 2 in the main text). The model assumes that YAP does not affect the vacuolation front speed v_{front}. The empirical measurements shown in the graph are the same as those presented in Figure 4A. The parameters used for the simulations are the same as the one used in Table 3 except normalized wild-type Yap level (0.2), normalized mutant Yap level (0.23), the maximum rate of vacuolation-induced cell growth (0.91 $\frac{\mu m}{h}$) and the maximum progenitor addition rate (35 $\frac{1}{h}$).

Development • Supplementary information

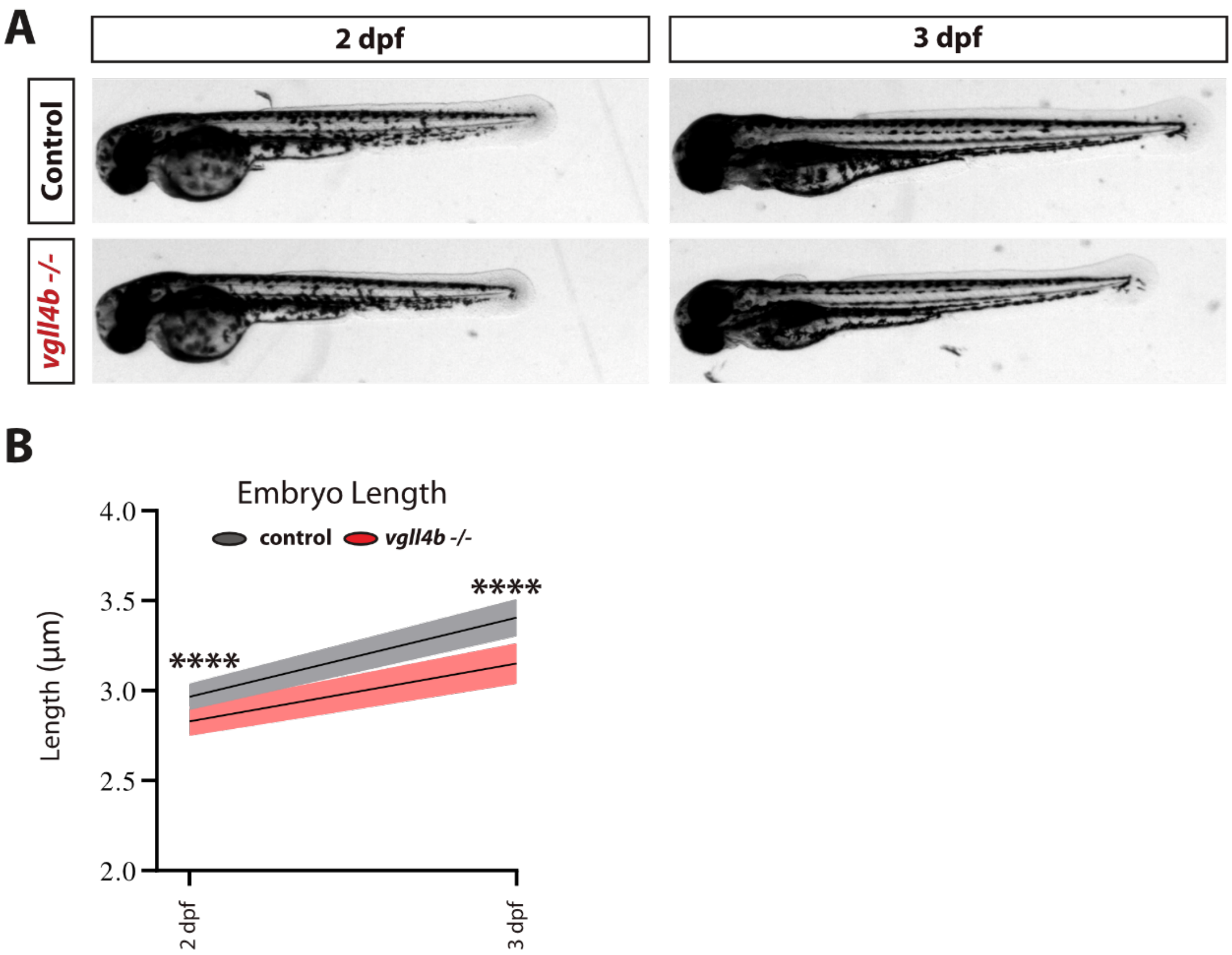

Fig. S6. Reduced axial length in *vgll4b* mutant embryos at later developmental stages.

A. Images of control and mutant embryos at 2 dpf and 3 dpf. **B.** Quantification of embryo length at 2 dpf (t-test; control n = 49, mutant n = 58; $p < 0.0001$) and 3 dpf (t-test; control n = 135, mutant n = 147; $p < 0.0001$).

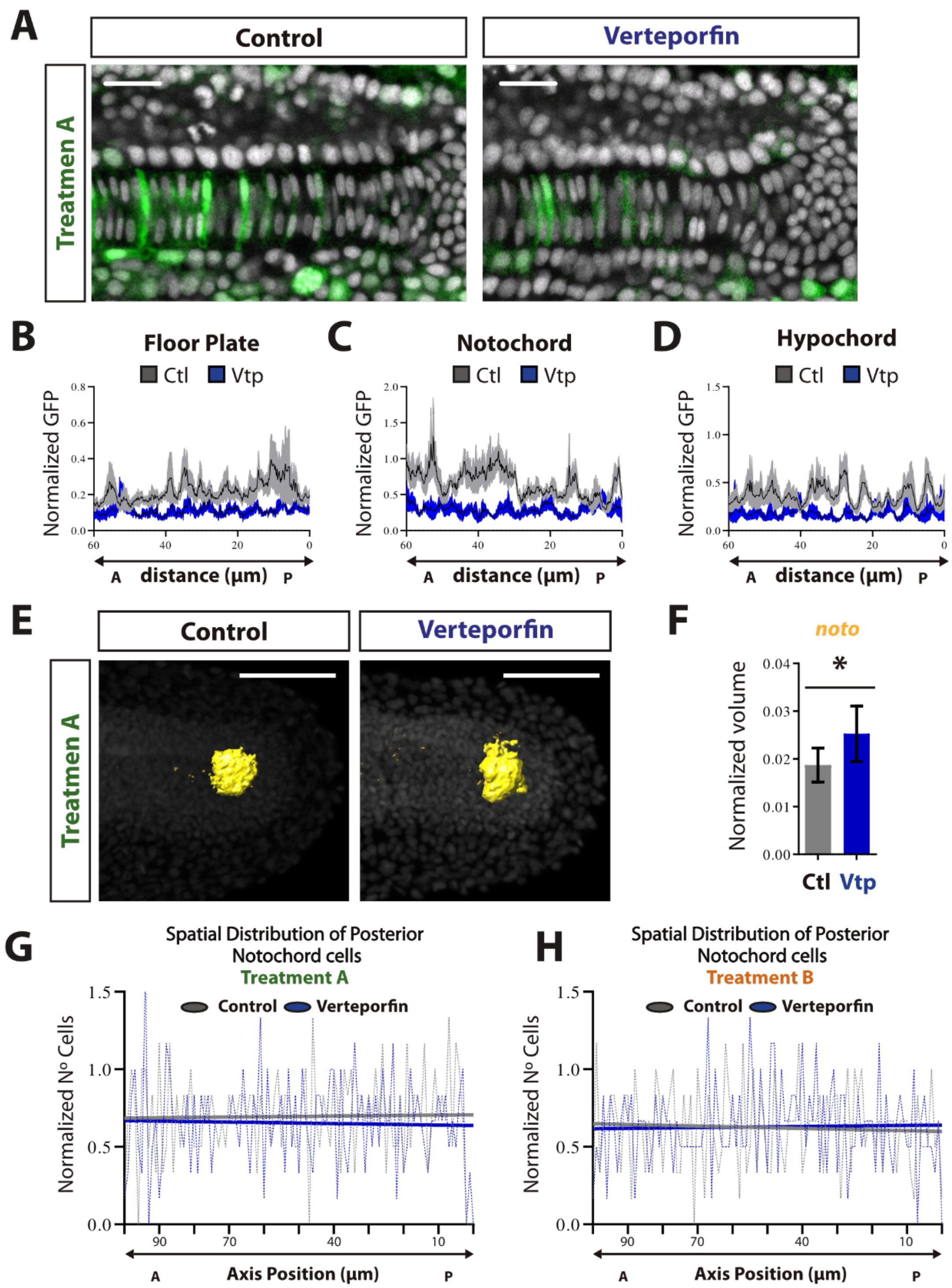

Fig. S7. Verteporfin treatment inhibits GFP expression in *Tg(4xGTIIC:GFP)* embryos. **A**. Representative confocal images of control and verteporfin-treated *Tg(4xGTIIC:GFP)* embryos (Treatment A) immunostained for GFP (scale bar: 100 µm). **B, C, D**. Quantification of normalized GFP intensity across the floor plate, notochord, and hypochord in control and verteporfin-treated embryos for Treatment A (control n = 6;

Development • Supplementary information

mutant n = 6). **E**. Volumes of *noto* expression from HCR staining in control and verteporfin-treated embryos for Treatment A (scale bar: 100 µm). **F**. Quantification of normalized *noto* expression volume (t-test; control n = 9, mutant n = 7; p = 0.0142). **G, H**. Graphical representation of the normalized number of notochord nuclei across the posterior axis in control and mutant embryos for Treatment A (control: n = 6, 422 cells; treated: n = 6, 396 cells) and Treatment B (control: n = 6, 378 cells; treated: n = 6, 380 cells). The region of interest spans 100 µm, beginning at the posterior end of the notochord, which corresponds to position 0 on the x-axis of the graph. The normalized number of notochord cells counted at each position along the axis is represented on the y-axis.

Table S1. Primers used for ISH probe generation

Gene	Primer Fw	Primer Rv
vgll4a	AGCATTTCCATCGCAGTCTG	CAGTCTGTCCAGGTGAGTGT
vgll4b	AACAACATCGGCATCCTGTG	ATGTGTGTGTTTGGAGAGGC
vgll4l	GCTGAGCGAGGAACAATTCA	TGACTATCAAACAACCTGCCT

Table S2. Primers used for qPCR experiments

Gene	Primer Fw	Primer Rv
ccn1	GACTGCGTGTATGAGAAGCG	TGGTTTCCTCTAGCTGCCAA
ccn2a	AACCTGTGGCTTGGGAATCT	GTTTGGAGACTCGTGGTGTG
axin2	TACCCTCGGACACTTCAAGG	CCATTCTGTCCACTTTGCCG
lfng	CGAGTCAGTTCTTGGGGTCT	CACACAGCCCAATACCACAC
cyyr1	CGTCTGCATGTGTGTGAAGA	TGTATGGAGGAGGACGGAGA

Development • Supplementary information

Table S3. Parameters corresponding to the mathematical model described by Eqs. 3 and 4 in the main text.

Parameter	Name	Value	Method
N_0	Initial number of cells	145	Experimentally obtained in this study.
$\frac{k}{\mu}$	Spring constant divided by the viscous drag coefficient	$100000\frac{1}{h}$	Estimated in this study.
v_0	Maximum vacuolation front speed	$250\ \frac{\mu m}{h}$	Estimated in this study.
J	Constant rate of vacuolation-induced cell growth	$0.73\ \frac{\mu m}{h}$	Experimentally obtained in this study.
T_p	Stop time for progenitor addition	9.5 h	Estimated in this study.
r_{p0}	Maximum progenitor addition rate	9.92 1/h	Estimated in this study.
Y	Normalized YAP expression level	0.72 (wild-type) and 0.84 (mutant)	Estimated in this study.
L_0	Initial tissue length	960.74 (wild-type) and 952.75 (mutant)	Experimentally obtained in this study.
t_{total}	Total time	19 h	Estimated in this study.
$\frac{F_{ext}}{\mu}$	Compression force due to progenitor addition divided by the viscous drag coefficient	$0.1\ \frac{\mu m}{h}$	Estimated in this study.
t_{start_vac}	Time at which vacuolation starts	0 h	Estimated in this study.

Development • Supplementary information
