# Supplementary material for: Coupling Vgll4b/Yap-regulated posterior cell addition with anterior vacuolation enables robust notochord elongation
Source: Development. 2026 Sep 14;153(17):dev205951. doi: 10.1242/dev.205951 (PMC13615686; doi:10.1242/dev.205951)
Supplement: Supplementary file 2 [file develop-153-205951_review_history.pdf]

Coupling Vgll4b/Yap regulated posterior cell addition with anterior vacuolation enables robust notochord elongation

Carlos Camacho-Macorra, Alberto Ceccarelli, Guillermo Serrano Najera, Dillan Saunders, Isabella Boesgaard, Osvaldo Chara and Benjamin Steventon
DOI: 10.1242/dev.205951

Editor: James Briscoe

Review timeline

Submission to Review Commons:	2 March 2026
Submission to Development	8 July 2026
Editorial Decision:	3 August 2026
First revision received:	7 August 2026
Accepted:	7 August 2026

Reviewer 1:

Evidence, reproducibility and clarity

This work focuses on zebrafish notochord morphogenesis during axial elongation. In particular it dissects the role of YAP signalling on regulating the balance between caudal cell addition with the cell enlargement occurring rostrally through vacuolation.

The article is timely to the field and includes several important experiments. The overall presentation and written style are good, citations are adequate and there is a clear effort to integrate experiments and mathematical modelling from the outset. The logic behind experiments is sound and the conclusion coherent (even if not totally unexpected given the literature): YAP affects progenitor addition which in turn changes packing, vacuolation and axis length. I just have a few points that could make the article clearer and more persuasive.

Major points

- Last section of results is difficult and confusing. After analysing vgll4b loss-of-function line, effectively over-activating YAP, the focus is on YAP inhibition using Verteporfin.
- Concerns on Verteporfin: the molecule has been widely used to module YAP, but there are also plenty of studies suggesting it is non-specific (also degrades YAP, has 14-4-3σ dependency and induces stress). I would consider an alternative: truncated TEAD, LATS over-expression or gain-of-function phosphomimetic versions of YAP.
- Presentation: regardless of point above, Verteporfin's role on YAP should be verified in the system. As such it is crucial to include: images of 4xGTIIC, noto and YAP stains after treatment. Only then inspect the effects on vacuolation and different treatments.
- In Fig 3F, noto HCR staining is taken as evidence for progenitor exhaustion/ faster depletion. Other scenarios would be possible without more direct demonstration. Evidence (either experimental or literature) that YAP is not involved in self-renewal or induction of these progenitors at these stages should be discussed.
- Individual datapoints in Fig 3C and 4D should be shown. Additional justification is needed as to why spinal cord is the best to benchmark displacement. Additionally looking at this with respect to mesoderm migration could capture another set of progenitors and behaviour/ displacements.
- Plotting vacuole area in Fig.4I vs A-P position (similar to plots 1H, 2F-H) could further strengthen the point of gradual (linear) vacuolation.

© 2026. Published by The Company of Biologists under the terms of the Creative Commons Attribution License (https://creativecommons.org/licenses/by/4.0/).

Minor points:

- Scheme of Fig1A could benefit from having the info of zebrafish timeline (hpf)
- Figure 3B, what was time 0?
- The authors should address whether Verteporfin-treated mutants are rescued or whether the compound overwhelms the genetic effect.
- Cell density is an elegant measure but quite abstract. A plot of cells detected at each AP position would be quite valuable to reinforce more cells are being added to a relatively constant area

Significance

Significance included above.

Reviewer 2:

Evidence, reproducibility and clarity

Summary

Camacho-Macorra et al. investigate the mechanisms of axis extension in zebrafish embryos, focusing on the notochord and its two key elongation processes: progenitor addition (occurring early and posteriorly) and vacuolization (occurring later and in an anterior to posterior sequence). The authors first develop a mathematical model to predict notochord elongation dynamics by integrating these processes. They demonstrate that the YAP signaling pathway is active in both the notochord and its progenitors during axial extension. Their analysis reveals that vgll4b, an inhibitor of YAP, is expressed in the same regions. Knockdown of vgll4b results in YAP hyperactivation in the notochord and posterior progenitor regions, leading to increased progenitor recruitment into the notochord and a reduction in the progenitor pool. The effects of this mutation on extension are most pronounced during the late phase, which is dominated by vacuolization. The authors observe smaller vacuoles in mutants during this phase. However, early (but not late) YAP inhibition decreases notochord cell density and increases vacuole size, suggesting that YAP primarily regulates notochord progenitor uptake, which indirectly affect vacuolization.

Major Comments

The authors propose that YAP activity mediates a long-range feedback mechanism linking posterior progenitor addition to anterior vacuolization. Two lines of evidence are presented to support this idea. First, there appears to be compensation for tissue length during Phase 2, when both progenitor addition and vacuolization occur. Second, temporal YAP inhibition experiments show that early, but not late, YAP inactivation affects both cell addition and vacuolization. While these observations are intriguing, they do not conclusively demonstrate spatial long-range coordination. Instead, the global decrease of vacuole size could be a simple delayed consequence of cell density increase or cell disorganization at the posterior end without involving a long-range feedback along AP axis. Claiming that such long-range feedback is taking place would require a more precise characterization and/or the identification of its nature (chemical, mechanical). Furthermore, there are several caveats with the interpretations of the claims cited above. The authors do not show quantification of vacuole area using notochord cell segmentation as described in Fig 1C in vgll4b mutants at stages when progenitor addition is increased. The slope of internuclear distances in Supplementary Figure 4A at 27 hours post-fertilization suggests that vacuolization is initially normal (similar to wt context in Fig 1H), arguing against an early defect in vacuolation dynamics along the Anterior to Posterior axis that could compensate for extra addition of progenitors. Finally, the timing of the analysis of the effect of Verteporfin treatments is unclear. According to the legend of Figure 4F, analyses for Treatment A (16-27 hpf) and Treatment B (27-38 hpf) were done at 24 hpf and 30 hpf, respectively. If this is the case, the 3-hour window for Treatment B may not allow sufficient time to reveal effects on vacuolization.
To strengthen the claim of long-range coupling, the authors could:
Provide direct measurements of vacuolization A-P dynamics/area during Phase 2, before the effect on notochord length in the mutant, to see if there is indeed a compensatory effect on notochord length for the additional accretion of notochord progenitors in the Vgll4b mutant.

© 2026. Published by The Company of Biologists under the terms of the Creative Commons Attribution License (https://creativecommons.org/licenses/by/4.0/).

Clarify the analysis timing of Treatment B to confirm that YAP inhibition during the vacuolization phase truly has no effect.
Additionally, as a non-specialist, I found the distinction between the two modeling hypotheses difficult to follow. Specifically, it is unclear why the first hypothesis assumes YAP affects vacuolation rate, while the second assumes it affects vacuolation front speed. It is also not intuitive how front speed can be independent of vacuolation rate, as one would expect that if cells form vacuoles more slowly, the front should progress more slowly as well. Therefore, it could be good to clarify these aspects of the modeling part.

Minor Comments

While the study is technically sound, a few areas could benefit from improved clarity or additional data.
An intriguing but puzzling finding is the reduction in the noto-expressing progenitor domain in vgll4b mutants, despite elevated YAP activity in progenitors. Intuitively, if YAP promotes progenitor maintenance or expansion, one might expect the noto+ domain to increase, not shrink. This paradox suggests that YAP may not only simply maintain progenitors but instead accelerates their differentiation or migration into the notochord (as stated in the manuscript and graphical abstract). Alternatively, YAP could only deplete the noto+ pool by driving premature entry into the notochord, though the lack of clear YAP upregulation in this domain would imply a non-cell autonomous role of YAP for this interpretation. The authors should discuss these possibilities more explicitly in the Discussion section and could consider including additional markers, such as proliferation assays or apoptosis markers, to clarify whether YAP affects progenitor proliferation, differentiation, or migration.
In Figure 2B, the YAP activity reporter signal in the posterior floor plate is not immediately obvious. The authors should consider providing higher-magnification insets.
In Figure 2C, the differences in tail shape between wild-type and mutant embryos are visually striking. If these differences have not been quantified or discussed, a brief comment in the text would be helpful.
Supplementary Figure 6 describes embryo length differences in mutants but does not include a representative image. Adding one would strengthen the phenotypic description.
Figure 1C is not cited in the text as not associated with a result, but just a description of the approach that is used later in Fig 4I
Finally, the authors might consider citing Michaud & Pourquié (2025) when presenting the role of hydrostatic pressure in axis elongation in the Introduction.

Significance

This study by Camacho-Macorra et al. presents a fascinating exploration of how YAP signaling and its inhibition by vgll4b coordinate progenitor addition and vacuolization during zebrafish notochord elongation. The work is well executed, with clear results and integration of mathematical modeling and experimental data. The findings shed new light on the molecular and mechanical regulation of axis extension, a fundamental process in vertebrate development. However, while the study is innovative and rigorously conducted, the central claim of "long-range coupling" between progenitor addition and vacuolization requires further substantiation. Addressing the points discussed below will make the study more convincing and accessible to developmental biologists and mechanobiologists alike.

reviewer expertise: developmental biologist specialised in morphogenesis

<u>Reviewer 3:</u>

Evidence, reproducibility and clarity

In the studies conducted by Camacho-Macorra et al., the authors examine the extension of the body axis is zebrafish, focusing on the notochord. They specifically compare timepoints where progenitor addition to the notochord and vacuolization are important to drive axis extension. They generate a simple mathematical model of notochord extension and show that it recapitulates observations in vivo where progenitor addition and vacuolation drive tissue elongation. They further perturb the system by showing that YAP activity is localized to the

© 2026. Published by The Company of Biologists under the terms of the Creative Commons Attribution License (https://creativecommons.org/licenses/by/4.0/).

midline progenitors of the notochord where when the competitive inhibitor of YAP vgll4b is perturbed it increases YAP signaling and results in increase progenitor addition to the notochord. They further describe a possible indirect-feedback mechanism linking YAP driven progenitor addition to the notochord with anterior vacuolation which when perturbed (i.e. increased YAP) results in reduced notochord elongation.

Major Comments: NA

Minor comments:
1.Figure 1B - please put the model equation in the figure or at least point out what variables of the equation refer to each part of the schematic.

2.Figure 1F - smooth line is misleading, please include individual embryo measurement points. This comment could be applied to several figures

3.Figure 2C/D - To make this manuscript more accessible to individuals who are not familiar with the anatomy of zebrafish tail, please include zoom in panels of the region of interest where arrows are pointing out increased YAP signaling in the floor plate and hypochord.

4.In discussion - "In vgll4b mutants, increased progenitor incorporation initially does not alter overall notochord length due to a buffering mechanism for natural variation in progenitor addition" - this is not directly tested in terms of buffering for variation and is an assumption. Please either cite a paper or reword

Significance

Overall, the logic and experiments conducted in these studies are well defined. However, the significance of the work is minimal and makes only a small contribution to the advancement of the field of developmental biology. Regardless, the studies are well done and worth publication.

Strengths:

- The study does a good job of incorporating and testing a computational model in a way that proves/disproves their hypothesis
- The manuscript is well written and follows a logical order, making it easy for readers to understand the main findings
- The study uses multiple routes of YAP inhibition (genetic and drug) to show effect on progenitor addition to the notochord and shortened body axis
- The discussion does aa very good job of giving the context of the study's results.

Weaknesses:

- The study is minimal and fails to illuminate the mechanism that connects progenitor addition to vacuolization, claiming only an indirect relationship with YAP signaling. However, this is admitted by the authors and not overstated

The study provides a minimal advancement to the field by investigating an unexplored area of zebrafish notochord extension. It provides a small step toward connecting mechanical/morphogenic mechanisms with signalling in zebrafish body axis extension.

The audience of this work is a specialized basic research group of developmental biology scientists. The research is of particular relevance to individuals studying zebrafish or axis elongation. While the authors make comparisons to other systems, due to the unique nature of the zebrafish body extension, this generates a narrow field of focus for the manuscript.

Author response to reviewers' comments

© 2026. Published by The Company of Biologists under the terms of the Creative Commons Attribution License (https://creativecommons.org/licenses/by/4.0/).

Reviewer #1 (Evidence, reproducibility and clarity (Required)):

This work focuses on zebrafish notochord morphogenesis during axial elongation. In particular it dissects the role of YAP signalling on regulating the balance between caudal cell addition with the cell enlargement occurring rostrally through vacuolation.

The article is timely to the field and includes several important experiments. The overall presentation and written style are good, citations are adequate and there is a clear effort to integrate experiments and mathematical modelling from the outset. The logic behind experiments is sound and the conclusion coherent (even if not totally unexpected given the literature): YAP affects progenitor addition which in turn changes packing, vacuolation and axis length. I just have a few points that could make the article clearer and more persuasive.

We thank the reviewer for these positive comments about our manuscript. We would like to reiterate the two main unexpected findings based on our results:
- While YAP mutants display a defective notochord (Kimelman et al., 2017; eLife) it has not been clear what specific role that YAP signalling is playing during notochord development. Therefore, the finding that Yap signalling plays a role in controlling the rate to notochord progenitor addition and represents a novel discovery.
- The observation that the notochord can buffer its elongation rate against an increased influx of progenitors is novel and counter intuitive. Our current understanding of tissue elongation depends on the central idea that the addition of progenitors directly impacts elongation rate. Here we show for the first time that this has minimal impact at the tissue level using the notochord as an example.

Major points

- Last section of results is difficult and confusing. After analysing vgll4b loss-of- function line, effectively over-activating YAP, the focus is on YAP inhibition using Verteporfin.
    - o Concerns on Verteporfin: the molecule has been widely used to module YAP, but there are also plenty of studies suggesting it is non-specific (also degrades YAP, has 14-4-3σ dependency and induces stress). I would consider an alternative: truncated TEAD, LATS over-expression or gain-of-function phosphomimetic versions of YAP.
    - o Presentation: regardless of point above, Verteporfin's role on YAP should be verified in the system. As such it is crucial to include: images of 4xGTIIC, noto and YAP stains after treatment. Only then inspect the effects on vacuolation and different treatments.

As suggested by the reviewer, we have added a supplementary figure validating the verteporfin treatment, including quantification of GFP reduction across the three tissues and quantification of notochord staining. We did not include Yap1 immunostaining data because the signal quality was insufficient for reliable analysis.
A simple over-expression experiment will not allow the spatial and temporal control required to test our hypothesis. Yap has a known function in gastrulation, so we need experiments that allow us to perturb Yap activity only at posterior body elongation stages. This has been achieved with the vgl4b experiments shown in the manuscript, as this gene is specifically expressed in the tailbud at these stages. In addition to the full verification of verteporfin's impact on YAP activity, we feel this is sufficient evidence to support our conclusions.

- In Fig 3F, noto HCR staining is taken as evidence for progenitor exhaustion/ faster depletion. Other scenarios would be possible without more direct demonstration. Evidence (either experimental or literature) that YAP is not involved in self-renewal or induction of these progenitors at these stages should be discussed.

We have concluded that the smaller volume of noto expressing cells is consistent with the faster depletion of the progenitor pool based on the direct observation of increased progenitor addition rate from photo-labelling experiments (Figure 3A,B). As suggested by the reviewer, we have now quantified cell divisions within the midline progenitor population and

© 2026. Published by The Company of Biologists under the terms of the Creative Commons Attribution License (https://creativecommons.org/licenses/by/4.0/).

found no significant differences between mutant and control embryos. These data have now been included in Supplementary figure 3.

- Individual datapoints in Fig 3C and 4D should be shown.
These data have now been added to the figures

Additional justification is needed as to why spinal cord is the best to benchmark displacement. Additionally looking at this with respect to mesoderm migration could capture another set of progenitors and behaviour/ displacements.
Photolabels within the pre-somitic mesoderm are difficult to interpret as the high amount of cell rearrangement in this tissue leads to a spreading out of the labelled clone in a manner that then makes it difficult to assess tissue displacement (see Figure 2D,E; Thomson et al., (2021) Cells and Development). In contrast, aprevious paper has shown that notochord-spinal cord displacements can be mapped in a reliable manner across the anterior-posterior axis which motivated our choice here (McLaren and Steventon (2021) Development).

- Plotting vacuole area in Fig.4I vs A-P position (similar to plots 1H, 2F-H) could further strengthen the point of gradual (linear) vacuolation.
As suggested by the reviewer, we have plotted vacuole area as a function of position for the verteporfin treatment experiments, and these data have now been included in Figure 5.

Minor points:
- Scheme of Fig1A could benefit from having the info of zebrafish timeline (hpf)
The scheme has been modified indicating zebrafish timeline
- Figure 3B, what was time 0?
Timepoints have now been included in the text and figure legend
- The authors should address whether Verteporfin-treated mutants are rescued or whether the compound overwhelms the genetic effect.
Given that verteporfin will impact Yap signalling in a global manner, whereas the vgl4b have a localised over-activation of Yap signalling, we think this experiment would be difficult to interpret and would likely be non-informative.
- Cell density is an elegant measure but quite abstract. A plot of cells detected at each AP position would be quite valuable to reinforce more cells are being added to a relatively constant area.
As suggested by the reviewer, we have now plotted these data for mutant and controls and also for verteporfin treatments. These data have now been included in supplementary figures 3 and 7.
Reviewer #1 (Significance (Required)):
Significance included above.

Reviewer #2 (Evidence, reproducibility and clarity (Required)):

Summary

Camacho-Macorra et al. investigate the mechanisms of axis extension in zebrafish embryos, focusing on the notochord and its two key elongation processes: progenitor addition (occurring early and posteriorly) and vacuolization (occurring later and in an anterior to posterior sequence). The authors first develop a mathematical model to predict notochord elongation dynamics by integrating these processes. They demonstrate that the YAP signaling pathway is active in both the notochord and its progenitors during axial extension. Their analysis reveals that vgll4b, an inhibitor of YAP, is expressed in the same regions. Knockdown of vgll4b results in YAP hyperactivation in the notochord and posterior progenitor regions, leading to increased progenitor recruitment into the notochord and a reduction in the progenitor pool. The effects of this mutation on extension are most pronounced during the late phase, which is dominated by vacuolization. The authors observe smaller vacuoles in mutants during this phase. However, early (but not late) YAP inhibition decreases notochord cell density and increases vacuole size, suggesting that YAP primarily regulates notochord progenitor uptake, which indirectly affect vacuolization.

Major Comments

© 2026. Published by The Company of Biologists under the terms of the Creative Commons Attribution License (https://creativecommons.org/licenses/by/4.0/).

The authors propose that YAP activity mediates a long-range feedback mechanism linking posterior progenitor addition to anterior vacuolization. Two lines of evidence are presented to support this idea. First, there appears to be compensation for tissue length during Phase 2, when both progenitor addition and vacuolization occur. Second, temporal YAP inhibition experiments show that early, but not late, YAP inactivation affects both cell addition and vacuolization. While these observations are intriguing, they do not conclusively demonstrate spatial long-range coordination. Instead, the global decrease of vacuole size could be a simple delayed consequence of cell density increase or cell disorganization at the posterior end without involving a long-range feedback along AP axis. Claiming that such long-range feedback is taking place would require a more precise characterization and/or the identification of its nature (chemical, mechanical).

We would like to thank this reviewer for this point, that we feel requires further clarification. As they suggest, the increased additional rate of posterior progenitors leads to a later impact on vacuolation, once these cells have reached more anterior parts of the body axis- creating an effective long-range feedback mechanism to link the two processes. However, this is not a direct propagation of a signal (mechanical or otherwise) across the length of the notochord, as may have been interpreted to be based on the previous framing of our conclusions.

We have modified the title of our manuscript to place less emphasis on the ‘long- range feedback’, and included an additional discussion paragraph to make this point clearer.

Furthermore, there are several caveats with the interpretations of the claims cited above. The authors do not show quantification of vacuole area using notochord cell segmentation as described in Fig 1C in vgll4b mutants at stages when progenitor addition is increased.

This is an important point highlighted by the reviewer. We have now included analysis at 24 hpf, where we do see a significant reduction in vacuole area within the anterior part of the notochord during the buffering phase in vgl4b mutants- consistent with our model that reduced anterior vacuolation compensates for increased progenitor addition rate during this phase of notochord elongation (Figure 4E).

The slope of internuclear distances in Supplementary Figure 4A at 27 hours post- fertilization suggests that vacuolization is initially normal (similar to wt context in Fig 1H), arguing against an early defect in vacuolation dynamics along the Anterior to Posterior axis that could compensate for extra addition of progenitors.

We have revised Supplementary Figure 4 to present a direct comparison between mutant and control embryos at each time point analyzed. This analysis shows that within the mid-trunk region of the notochord, differences in cell size first emerge at the developmental stage when vacuolation becomes the primary driver of axis elongation. In addition, we observe a progressive decoupling of the scaling relationship in mutant embryos over time. As mentioned above- there is a significant difference in vacuole size within more anterior regions at 22.5 hpf that is consistent with the model that this is buffering against increase posterior addition.

Finally, the timing of the analysis of the effect of Verteporfin treatments is unclear. According to the legend of Figure 4F, analyses for Treatment A (16-27 hpf) and Treatment B (27-38 hpf) were done at 24 hpf and 30 hpf, respectively. If this is the case, the 3-hour window for Treatment B may not allow sufficient time to reveal effects on vacuolization.

We agree that the information regarding the verteporfin experiments was not clearly presented in the original figure, and we have therefore revised the schematic accordingly.

To strengthen the claim of long-range coupling, the authors could:

Provide direct measurements of vacuolization A-P dynamics/area during Phase 2, before the effect on notochord length in the mutant, to see if there is indeed a compensatory effect on notochord length for the additional accretion of notochord progenitors in the Vgll4b mutant.

© 2026. Published by The Company of Biologists under the terms of the Creative Commons Attribution License (https://creativecommons.org/licenses/by/4.0/).

As suggested by the reviewer, we have added an earlier time point to the A–P area dynamics plot in phase 2, corresponding to a stage at which the effect on notochord length in the mutant is not yet detectable. At this stage, we observed no difference in vacuole area between mutants and controls. We have also included an earlier time point analysis in the anterior region of the axis, which shows a similar cell size difference to that observed later in a more posterior region (Figure 4F; see above response).

Clarify the analysis timing of Treatment B to confirm that YAP inhibition during the vacuolization phase truly has no effect.

This has now been clarified.

Additionally, as a non-specialist, I found the distinction between the two modeling hypotheses difficult to follow. Specifically, it is unclear why the first hypothesis assumes YAP affects vacuolation rate, while the second assumes it affects vacuolation front speed. It is also not intuitive how front speed can be independent of vacuolation rate, as one would expect that if cells form vacuoles more slowly, the front should progress more slowly as well. Therefore, it could be good to clarify these aspects of the modeling part.

We thank the reviewer for this comment and apologise for the lack of clarity in our description of the model. In our framework, the cell size profile along the AP axis of the notochord is governed by two distinct processes: (i) the addition of progenitors at the posterior tip, and (ii) vacuolation, which increases cell size and proceeds from anterior to posterior. We model the latter as a propagating wave with velocity vf, such that cells begin to vacuolate when the wave front reaches their position.

Importantly, in the model these two aspects of vacuolation are decoupled: the front velocity vf determines when a given cell starts vacuolating, whereas the vacuolation rate J determines how fast the cell increases in size once the process has started. Biologically, this corresponds to distinguishing between the propagation of a trigger or competence signal along the tissue, and the execution of vacuole growth within each cell. Our reasoning was that they need not be strictly proportional: a signalling wave could propagate at a given speed even if the downstream cellular response is slower or faster.

This is why we considered two alternative hypotheses: either YAP modulates the propagation of the vacuolation front (affecting vf), or it modulates the growth dynamics within each cell (affecting J). Our quantitative comparison with the experimental data supports the former scenario. This has now been clarified in the main text.

Minor Comments

While the study is technically sound, a few areas could benefit from improved clarity or additional data.

An intriguing but puzzling finding is the reduction in the noto-expressing progenitor domain in vgll4b mutants, despite elevated YAP activity in progenitors. Intuitively, if YAP promotes progenitor maintenance or expansion, one might expect the noto+ domain to increase, not shrink. This paradox suggests that YAP may not only simply maintain progenitors but instead accelerates their differentiation or migration into the notochord (as stated in the manuscript and graphical abstract). Alternatively, YAP could only deplete the noto+ pool by driving premature entry into the notochord, though the lack of clear YAP upregulation in this domain would imply a non-cell autonomous role of YAP for this interpretation. The authors should discuss these possibilities more explicitly in the Discussion section and could consider including additional markers, such as proliferation assays or apoptosis markers, to clarify whether YAP affects progenitor proliferation, differentiation, or migration.

As also suggested by the reviewer, we have included a cell proliferation analysis in Supplementary Figure 3 and have revised the Discussion section accordingly.

© 2026. Published by The Company of Biologists under the terms of the Creative Commons Attribution License (https://creativecommons.org/licenses/by/4.0/).

In Figure 2B, the YAP activity reporter signal in the posterior floor plate is not immediately obvious. The authors should consider providing higher-magnification insets.

As suggested by the reviewer, we have included higher-magnification insets in Figure 2

In Figure 2C, the differences in tail shape between wild-type and mutant embryos are visually striking. If these differences have not been quantified or discussed, a brief comment in the text would be helpful.

We did not see a consistent impact on the morphology of the posterior body, this has now been clarified in the main text.

Supplementary Figure 6 describes embryo length differences in mutants but does not include a representative image. Adding one would strengthen the phenotypic description.

As suggested by the reviewer, we have modified Supplementary Figure 6

Figure 1C is not cited in the text as not associated with a result, but just a description of the approach that is used later in Fig 4I

We have modified the text to include the appropriate figure reference.

Finally, the authors might consider citing Michaud & Pourquié (2025) when presenting the role of hydrostatic pressure in axis elongation in the Introduction.

We have now modified the text to include this citation which we agree is relevant to this work.

Reviewer #2 (Significance (Required)):

This study by Camacho-Macorra et al. presents a fascinating exploration of how YAP signaling and its inhibition by vgll4b coordinate progenitor addition and vacuolization during zebrafish notochord elongation. The work is well executed, with clear results and integration of mathematical modeling and experimental data. The findings shed new light on the molecular and mechanical regulation of axis extension, a fundamental process in vertebrate development. However, while the study is innovative and rigorously conducted, the central claim of "long-range coupling" between progenitor addition and vacuolization requires further substantiation. Addressing the points discussed below will make the study more convincing and accessible to developmental biologists and mechanobiologists alike.
reviewer expertise: developmental biologist specialised in morphogenesis

Reviewer #3 (Evidence, reproducibility and clarity (Required)):

In the studies conducted by Camacho-Macorra et al., the authors examine the extension of the body axis is zebrafish, focusing on the notochord. They specifically compare timepoints where progenitor addition to the notochord and vacuolization are important to drive axis extension. They generate a simple mathematical model of notochord extension and show that it recapitulates observations in vivo where progenitor addition and vacuolation drive tissue elongation. They further perturb the system by showing that YAP activity is localized to the midline progenitors of the notochord where when the competitive inhibitor of YAP vgll4b is perturbed it increases YAP signaling and results in increase progenitor addition to the notochord. They further describe a possible indirect-feedback mechanism linking YAP driven progenitor addition to the notochord with anterior vacuolation which when perturbed (i.e. increased YAP) results in reduced notochord elongation.

Major Comments: NA

Minor comments:
1. Figure 1B - please put the model equation in the figure or at least point out what variables of the equation refer to each part of the schematic.

© 2026. Published by The Company of Biologists under the terms of the Creative Commons Attribution License (https://creativecommons.org/licenses/by/4.0/).

As suggested by the reviewer, we have modified the scheme in Figure 1
2. Figure 1F - smooth line is misleading, please include individual embryo measurement points. This comment could be applied to several figures
We agree with the reviewer that the graphs in the original manuscript could be improved, and we have therefore modified all figures to better represent data dispersion within each group.
3. Figure 2C/D - To make this manuscript more accessible to individuals who are not familiar with the anatomy of zebrafish tail, please include zoom in panels of the region of interest where arrows are pointing out increased YAP signaling in the floor plate and hypochord.
As suggested by the reviewer, we have included higher-magnification insets in Figure 2
4. In discussion - "In vgll4b mutants, increased progenitor incorporation initially does not alter overall notochord length due to a buffering mechanism for natural variation in progenitor addition" - this is not directly tested in terms of buffering for variation and is an assumption. Please either cite a paper or reword

This point has been clarified in the revised discussion.

Reviewer #3 (Significance (Required)):
Overall, the logic and experiments conducted in these studies are well defined. However, the significance of the work is minimal and makes only a small contribution to the advancement of the field of developmental biology.
Regardless, the studies are well done and worth publication. Strengths:
-The study does a good job of incorporating and testing a computational model in a way that proves/disproves their hypothesis
-The manuscript is well written and follows a logical order, making it easy for readers to understand the main findings
-The study uses multiple routes of YAP inhibition (genetic and drug) to show effect on progenitor addition to the notochord and shortened body axis
-The discussion does aa very good job of giving the context of the study's results. Weaknesses:
-The study is minimal and fails to illuminate the mechanism that connects progenitor addition to vacuolization, claiming only an indirect relationship with YAP signaling. However, this is admitted by the authors and not overstated
The study provides a minimal advancement to the field by investigating an unexplored area of zebrafish notochord extension. It provides a small step toward connecting mechanical/morphogenic mechanisms with signalling in zebrafish body axis extension.
The audience of this work is a specialized basic research group of developmental biology scientists. The research is of particular relevance to individuals studying zebrafish or axis elongation. While the authors make comparisons to other systems, due to the unique nature of the zebrafish body extension, this generates a narrow field of focus for the manuscript.

We have previously discussed the uniqueness of zebrafish posterior body elongation in light of critical differences in the degree to which posterior growth from self-renewing tailbud progenitor populations contribute to the mechanisms of axis elongation (Sambasivan and Steventon (2021) Frontiers in cell and dev. Biol; Steventon and Martinez Arias (2017) Developmental Biology). Here too, we think zebrafish provide an important system to explore differences in the mechanisms that drive notochord elongation, and we envisage that this study will provoke a similar cross species comparison that takes into account differences in the relative timing of progenitor addition and anterior notochord expansion (that occurs much later in amniotes, for example). It is only by considering these species-specific differences across experimental organisms that we can arrive at the fundamental principles that drive developmental processes, and how evolution has acted upon these to drive change in adult body plans. We therefore respectfully disagree with the review about the scope and importance of this work for these reasons.

In addition, we feel that the principles by which dynamic processes are coupled across an organ are broadly applicable and will illuminate further research into understanding organ growth control.

© 2026. Published by The Company of Biologists under the terms of the Creative Commons Attribution License (https://creativecommons.org/licenses/by/4.0/).

Original submission

First decision letter

MS ID#: dev.205951

MS TITLE: Vgll4b controls cell addition rate via Yap signalling and couples with anterior vacuolation to provide robust notochord elongation

AUTHORS: Carlos Camacho-Macorra, Alberto Ceccarelli, Guillermo Serrano Najera, Dillan Saunders, Isabella Boesgaard, Osvaldo Chara and Benjamin Steventon

Dear Dr Steventon,

Thank you for sending your manuscript to Development through Review Commons.

I have now received all the referees reports on the above manuscript, and have reached a decision. The referees' comments are appended below.

The overall evaluation is positive and we would like to publish a revised manuscript in Development, provided that the referees' comments can be satisfactorily addressed. The remaining points are about presentation and clarification rather than new experiments. Please show the individual datapoints in Fig. 3C and 4D, which appear unchanged from the previous version. The x-axis of Fig. 4F should also reflect the region actually quantified at 22.5 hpf, so that the anterior position of the phenotype is clear rather than plotted on the same scale as the later time points, and in Supplementary Fig. 3B-D please explain why the phospho-histone H3 counts are centred on zero and how the negative values should be read. Please clarify why the difference in vacuole area along the anteroposterior axis is significant at 22.5 hpf but not at 24 hpf. The argument that the earlier appearance of smaller anterior vacuoles shows that increased cell number does not affect the initiation of vacuolisation is hard to follow as written. On verteporfin, please explain why the drug alone was informative but uninterpretable in combination with the mutant. Finally, please address the point about the wider YAP literature on axial development, including axis truncation and effects on tailbud and notochord precursors.‹ Please attend to all of the reviewers' comments in your revised manuscript and detail them in your point-by-point response. If you do not agree with any of their criticisms or suggestions explain clearly why this is so. If it would be helpful, you are welcome to contact us to discuss your revision in greater detail. Please send us a point-by-point response indicating your plans for addressing the referees' comments, and we will look over this and provide further guidance.

Reviewer 1

In this manuscript from Camacho-Macorra et al., the authors investigate the role of YAP signaling during axis extension, using the zebrafish notochord as a model system to explore how tissue growth and differentiation are coordinated. They use mathematical models to predict growth rates that result from the combination of progenitor addition and vacuolization as the notochord moves through distinct mechanical phases of its extension. Both the mathematical model and empirical measurements of the embryo show a linear growth rate across multiple phases of notochord elongation. They examine YAP signaling as a possible regulatory mechanism to control linear notochord elongation across phases and examine YAP and its inhibitor VGLL4 in the developing zebrafish notochord using a YAP activity reporter zebrafish line. Fish mutant for vgll4 show increased activation of YAP in the notochord progenitor niche that results in increased progenitor addition to the notochord when compared to controls. The authors then go on to propose that YAP activity controls the progenitor addition and vacuolation front speed but not the rate of vacuolation and experimentally test this with computational models supported by empirical observations. Finally, they show that chemically disrupting YAP activity at later stages when notochord extension relies only on vacuolation does not affect the posterior notochord cell density or vacuole area while YAP disruption at the progenitor addition stage disrupted both. Collectively, this work

© 2026. Published by The Company of Biologists under the terms of the Creative Commons Attribution License (https://creativecommons.org/licenses/by/4.0/).

demonstrates an indirect feedback loop between notochord progenitor addition, controlled by YAP activity, and subsequent vacuolation to control notochord elongation.

Overall, this study was logically executed and the computational and experimental results well explained. It tackles an interesting problem of how embryonic tissues control their growth as progenitor pools are depleted and the mechanical mechanisms of growth change. They do a good job of addressing reviewer comments to strengthen the paper and provide increased evidence of a long-ranged coupling mechanism between YAP control over progenitor addition and vacuolation to ensure a linear growth across phases of axis elongation. In particular, drawing attention to the point that counter intuitively increased progenitor addition does not drive axis extension, but rather, due to this feedback disrupts a balanced scaling system resulting in shortened axis. This finding in itself is novel and of interest to the larger developmental biology field and the study of morphogenesis. I congratulate the authors on a well executed and significant manusciprt and recommend acceptance.

Reviewer 2

The revised manuscript and the authors' responses to my comments are fully satisfactory. I therefore support publication of the manuscript in Development and congratulate the authors on their excellent work.

I have only a few minor comments regarding points that could be clarified:

It is unclear why the difference in vacuole area along the anteroposterior axis between control and mutant embryos is significant at 22.5 hpf (Figure 4E-F), but no longer significant at 24 hpf (Figure 4D and Supplementary Figure 4C) when the measurements at 22.5 hpf have been performed in a more anterior region. The authors' interpretation—that the earlier presence of smaller vacuoles in the anterior region suggests that the increased cell number in the mutant does not affect the initiation of vacuolization—is difficult to follow. A logical step or a piece of information may be missing, and this point could be clarified.
The x-axis of the graph in Figure 4F is a bit confusing. According to the figure legend, the quantification at 22.5 hpf was performed over a region extending 400 μm anterior to the yolk extension. However, the graph uses the same scale as those for the other time points, from −200 to +200 μm relative to the yolk extension. Since position 0 does not appear to refer to the yolk extension at 22.5 hpf, the anterior location of the phenotype could be indicated more clearly, and an axis extending, for example, from −400 to 0 μm might be more appropriate.

In Supplementary Figure 3B-D, it is unclear why the quantification of phospho-histone H3-positive cells is centred on zero. I understand that very few cells are dividing and that values close to zero are therefore expected. However, the plots also display negative values, which are difficult to interpret and could be clarified.

Reviewer 3

The authors now added additional information on verteporfin treatment, cell cycle data and include new plots and justifications for some of their decisions, e.g. displacement measures. I believe these strengthen the manuscript and make it more compelling for readers.

This is probably beyond scope, but this reviewer struggles to understand why verteporfin alone was good enough for the inhibition data but would be "difficult to interpret and non-informative" in combination with the mutant. My initial concern about verteporfin as a possible disruptor of lysosomal function stands. I would also point out that it is not clear what is meant by YAP "signal quality was insufficient" in the verteporfin-treated embryos as it appears OK in the untreated controls Fig2B.\

My final comment would be to engage more with other parts of the YAP literature that could be at play in this process. E.g.
YAP-/- mice have a truncated axis. Effects on tailbud and notochord precursors are also looked at in https://doi.org/10.1128/MCB.26.1.77-87.2006
YAP1 KO in hESCs also leads to mesoderm defects in https://doi.org/10.1016/j.stemcr.2021.12.012

© 2026. Published by The Company of Biologists under the terms of the Creative Commons Attribution License (https://creativecommons.org/licenses/by/4.0/).

First revision

Author response to reviewers' comments

Reviewer 1: In this manuscript from Camacho-Macorra et al., the authors investigate the role of YAP signaling during axis extension, using the zebrafish notochord as a model system to explore how tissue growth and differentiation are coordinated. They use mathematical models to predict growth rates that result from the combination of progenitor addition and vacuolization as the notochord moves through distinct mechanical phases of its extension. Both the mathematical model and empirical measurements of the embryo show a linear growth rate across multiple phases of notochord elongation. They examine YAP signaling as a possible regulatory mechanism to control linear notochord elongation across phases and examine YAP and its inhibitor VGLL4 in the developing zebrafish notochord using a YAP activity reporter zebrafish line. Fish mutant for vgll4 show increased activation of YAP in the notochord progenitor niche that results in increased progenitor addition to the notochord when compared to controls. The authors then go on to propose that YAP activity controls the progenitor addition and vacuolation front speed but not the rate of vacuolation and experimentally test this with computational models supported by empirical observations. Finally, they show that chemically disrupting YAP activity at later stages when notochord extension relies only on vacuolation does not affect the posterior notochord cell density or vacuole area while YAP disruption at the progenitor addition stage disrupted both. Collectively, this work demonstrates an indirect feedback loop between notochord progenitor addition, controlled by YAP activity, and subsequent vacuolation to control notochord elongation.

Overall, this study was logically executed and the computational and experimental results well explained. It tackles an interesting problem of how embryonic tissues control their growth as progenitor pools are depleted and the mechanical mechanisms of growth change. They do a good job of addressing reviewer comments to strengthen the paper and provide increased evidence of a long-ranged coupling mechanism between YAP control over progenitor addition and vacuolation to ensure a linear growth across phases of axis elongation. In particular, drawing attention to the point that counter intuitively increased progenitor addition does not drive axis extension, but rather, due to this feedback disrupts a balanced scaling system resulting in shortened axis. This finding in itself is novel and of interest to the larger developmental biology field and the study of morphogenesis. I congratulate the authors on a well executed and significant manusciprt and recommend acceptance.

We thank the reviewer for these positive comments, and again for the previous set of suggestions that enabled us to improve the clarity of our manuscript.

Reviewer 2: The revised manuscript and the authors' responses to my comments are fully satisfactory. I therefore support publication of the manuscript in Development and congratulate the authors on their excellent work.

I have only a few minor comments regarding points that could be clarified:

- It is unclear why the difference in vacuole area along the anteroposterior axis between control and mutant embryos is significant at 22.5 hpf (Figure 4E-F), but no longer significant at 24 hpf (Figure 4D and Supplementary Figure 4C) when the measurements at 22.5 hpf have been performed in a more anterior region. The authors' interpretation—that the earlier presence of smaller vacuoles in the anterior region suggests that the increased cell number in the mutant does not affect the initiation of vacuolization—is difficult to follow. A logical step or a piece of information may be missing, and this point could be clarified.

We thank the reviewer for highlighting this point and allowing us to clarify the spatial and temporal logic of our vacuolation quantifications. Throughout the paper, we have focused on the central region of the axis, centered at the yolk extension using a 400 μm ROI. Within this central region, 24

© 2026. Published by The Company of Biologists under the terms of the Creative Commons Attribution License (https://creativecommons.org/licenses/by/4.0/).

hpf represents the earliest time point at which individual vacuoles can be reliably identified and segmented across the ROI; however, because vacuolation in this mid-axial zone is only just initiating at 24 hpf, vacuoles are extremely small in both genotypes and do not yet display a statistically significant difference in size. To specifically assess whether vacuolation is affected at even earlier stages, we conducted a targeted analysis at 22.5 hpf by shifting our measurement ROI to the anterior region of the notochord. Because notochord cell vacuolation follows a wave-like anterior-to-posterior progression, cells in this anterior region undergo vacuolation earlier than those in the central region. Consequently, at 22.5 hpf in the anterior region, vacuole expansion is already well underway, allowing us to detect a significant reduction in vacuole area in *vgll4b* mutants compared to controls. As development proceeds and the wave of vacuolation reaches the central region, this same significant reduction in vacuole area becomes evident at 27 hpf and remains significant at later stages.

Thus, the anterior measurement at 22.5 hpf demonstrates that vacuole initiation itself is not blocked, but rather that as vacuoles expand along the A–P axis, their overall area is constrained in the mutant. This is an important point to support our suggestion that anterior inhibition of vacuolation may be compensating for the increased additional of cells to enable the elongation rate of the notochord to remain the same as controls up until 27hpf (Figure 4A)- as mentioned in the previous round of comments. We have now clarified these points in the revised version of the manuscript.

- The x-axis of the graph in Figure 4F is a bit confusing. According to the figure legend, the quantification at 22.5 hpf was performed over a region extending 400 μm anterior to the yolk extension. However, the graph uses the same scale as those for the other time points, from −200 to +200 μm relative to the yolk extension. Since position 0 does not appear to refer to the yolk extension at 22.5 hpf, the anterior location of the phenotype could be indicated more clearly, and an axis extending, for example, from −400 to 0 μm might be more appropriate.

We are grateful to the reviewer for pointing this out and fully agree that adjusting the spatial coordinates improves the clarity of Figure 4F. In accordance with the reviewer's suggestion, we have modified the x-axis of the graph to display a range from −400 to 0 μm relative to the yolk extension, accurately reflecting the anterior region quantified at 22.5 hpf. We have updated Figure 4F and adjusted the figure legend accordingly.

- In Supplementary Figure 3B-D, it is unclear why the quantification of phospho-histone H3-positive cells is centred on zero. I understand that very few cells are dividing and that values close to zero are therefore expected. However, the plots also display negative values, which are difficult to interpret and could be clarified.

We thank the reviewer for this observation and completely agree that displaying violin plots for this dataset is not optimal and causes unnecessary confusion. To resolve this issue and improve clarity, we have replaced the original violin plots in Supplementary Figure 3B–D with standard bar plots showing mean ± standard deviation (SD). This representation accurately reflects the discrete counting nature of the data, sets the baseline clearly at zero, and avoids displaying non-physiological negative values. Accordingly, we have updated the corresponding figure legend to reflect the change in graph type.

Reviewer 3:

The authors now added additional information on verteporfin treatment, cell cycle data and include new plots and justifications for some of their decisions, e.g. displacement measures. I believe these strengthen the manuscript and make it more compelling for readers.

- This is probably beyond scope, but this reviewer struggles to understand why verteporfin alone was good enough for the inhibition data but would be "difficult to interpret and non-informative" in combination with the mutant.

We thank the reviewer for allowing us to clarify both our experimental logic and the technical details regarding YAP immunofluorescence.

© 2026. Published by The Company of Biologists under the terms of the Creative Commons Attribution License (https://creativecommons.org/licenses/by/4.0/).

Our primary objective in this study is to elucidate the specific role of YAP activity within midline progenitor cells during notochord morphogenesis. To address this, we used two complementary, single-variable approaches: genetic upregulation using *vgll4b* loss-of-function mutants to elevate YAP activity, and pharmacological downregulation via verteporfin treatment in wild-type embryos to transiently inhibit YAP activity. While *vgll4b* mutants exhibit elevated YAP activity in midline progenitors, *vgll4b* loss can also alter YAP signaling across multiple adjacent tissues. Combining verteporfin with *vgll4b* mutants would introduce systemic, multi-tissue YAP suppression on top of a broad mutant background, creating confounding systemic effects that would obscure the specific cell-autonomous or local tissue dynamics of midline progenitors. Furthermore, because verteporfin acts downstream by blocking YAP–TEAD interactions rather than directly replacing Vgll4b binding, we thought that a double manipulation would yield complex chemical-genetic interaction profiles that are difficult to interpret cleanly.

- My initial concern about verteporfin as a possible disruptor of lysosomal function stands.

Regarding the reviewer's concern about off-target lysosomal effects of verteporfin, while high concentrations or prolonged treatments in cell culture can affect lysosomal function, our short temporal treatment windows were specifically validated using our transgenic YAP reporter line and *noto* expression volume. The observed opposing phenotypes between *vgll4b* mutants and verteporfin-treated embryos, strongly support that verteporfin acts primarily via YAP pathway inhibition in this specific tissue context. Nevertheless, we have highlighted the potential caveat of using verteporfin within a revised discussion.

- I would also point out that it is not clear what is meant by YAP "signal quality was insufficient" in the verteporfin-treated embryos as it appears OK in the untreated controls Fig2B.

We also wish to clarify our statement regarding YAP "signal quality". Whole-mount Yap1 immunostaining in control embryos was sufficient to confirm the spatial localization of Yap1 protein within the notochord and midline progenitor niche, validating previous descriptive findings in zebrafish. However, antibody-based Yap1 immunofluorescence exhibited high background-to-noise ratios and variability in staining depth, which precluded reliable fluorescence intensity quantification across treatment groups. Furthermore, verteporfin disrupts the physical interaction between YAP and TEAD transcription factors without necessarily altering total Yap1 protein levels or nuclear/cytoplasmic localization. For these reasons, rather than relying on Yap1 antibody intensity to quantify verteporfin efficacy, we evaluated pathway inhibition using functional and transcriptional readouts, specifically the quantification of GFP intensity in the reporter line and the measurement of *noto* mRNA expression volume via HCR. These metrics provided a precise, quantifiable measure of YAP transcriptional activity in response to drug treatment.

- My final comment would be to engage more with other parts of the YAP literature that could be at play in this process. E.g.
  YAP-/- mice have a truncated axis. Effects on tailbud and notochord precursors are also looked at in https://doi.org/10.1128/MCB.26.1.77-87.2006
  YAP1 KO in hESCs also leads to mesoderm defects in https://doi.org/10.1016/j.stemcr.2021.12.012

Added to the introduction

Second decision letter

MS ID#: dev.205951R1

MS TITLE: Coupling Vgll4b/Yap regulated posterior cell addition with anterior vacuolation enables robust notochord elongation

© 2026. Published by The Company of Biologists under the terms of the Creative Commons Attribution License (https://creativecommons.org/licenses/by/4.0/).

AUTHORS: Carlos Camacho-Macorra, Alberto Ceccarelli, Guillermo Serrano Najera, Dillan Saunders, Isabella Boesgaard, Osvaldo Chara and Benjamin Steventon

Dear Dr Steventon,

I am happy to tell you that your manuscript has been accepted for publication in Development, pending our standard publication integrity checks.

© 2026. Published by The Company of Biologists under the terms of the Creative Commons Attribution License (https://creativecommons.org/licenses/by/4.0/).
